# Radiohistogenomics of pediatric low-grade neuroepithelial tumors

**DOI:** 10.1007/s00234-021-02691-1

**Published:** 2021-03-29

**Authors:** Asim K. Bag, Jason Chiang, Zoltan Patay

**Affiliations:** 1 Department of Diagnostic Imaging, St. Jude Children’s Research Hospital, 262 Danny Thomas Place, Mail Stop 220, Memphis, TN 38105 USA; 2 Department of Pathology, St. Jude Children’s Research Hospital, Memphis, TN USA

**Keywords:** Pediatric low-grade glioma (PLGG), Pediatric low-grade neuroepithelial tumors (PLGNTs), MAPK, MRI, Imaging, Radiology, KIAA1549-BRAF fusion, BRAF p.V600E

## Abstract

**Purpose:**

In addition to histology, genetic alteration is now required to classify many central nervous system (CNS) tumors according to the most recent World Health Organization CNS tumor classification scheme. Although that is still not the case for classifying pediatric low-grade neuroepithelial tumors (PLGNTs), genetic and molecular features are increasingly being used for making treatment decisions. This approach has become a standard clinical practice in many specialized pediatric cancer centers and will likely be more widely practiced in the near future. This paradigm shift in the management of PLGNTs necessitates better understanding of how genetic alterations influence histology and imaging characteristics of individual PLGNT phenotypes.

**Methods:**

The complex association of genetic alterations with histology, clinical, and imaging of each phenotype of the extremely heterogeneous PLGNT family has been addressed in a holistic approach in this up-to-date review article. A new imaging stratification scheme has been proposed based on tumor morphology, location, histology, and genetics. Imaging characteristics of each PLGNT entity are also depicted in light of histology and genetics.

**Conclusion:**

This article reviews the association of specific genetic alteration with location, histology, imaging, and prognosis of a specific tumor of the PLGNT family and how that information can be used for better imaging of these tumors.

## Introduction

Pediatric low-grade neuroepithelial tumors (PLGNTs) are the most common central nervous system (CNS) tumors in children, and they constitute approximately 30% of all brain tumors in children [1]. PLGNTs are World Health Organization (WHO) grade I or grade II low-grade gliomas and glioneuronal tumors that arise throughout the neuraxis [2, 3]. Tumors of the PLGNT family have been classified by histology, even in the most recent WHO CNS tumor classification system. Accordingly, the classical radiology approach has been to describe imaging appearances of a specific histologic entity. This approach, however, is rapidly changing.

In the last two decades, tremendous advancements have been made in understanding the genetic basis of PLGNTs. The profound impact of genetics on biology, morphology, and prognosis of individual PLGNT phenotypes is now being gradually unraveled [4]. We now know that specific genetic and epigenetic architectures have a predilection for specific age groups, specific brain areas, and specific morphological variants of PLGNTs [3, 5, 6]. Uncovering these features has led to better characterization and classification of these entities, specifically in tumors with overlapping histologic phenotypes [4, 5]. Although genetic alteration data are still not required for classifying PLGNTs according to the most recent WHO CNS tumor classification system [3], nevertheless, genetic and molecular features are increasingly being used to make treatment decisions for patients with PLGNTs [4, 5, 7]. This approach has become a standard clinical practice in many specialized pediatric cancer centers and will likely be more widely practiced in the near future. This paradigm shift in managing PLGNTs necessitates a better understanding of how genetic alterations influence histology and imaging of individual PLGNT phenotypes. In this review, we discuss recent advances in our understanding of the genetic underpinnings of PLGNTs and how those genetic features can be used in imaging for better imaging of the tumors of the PLGNT family.

## The genetic landscape of PLGNTs

**Fig. 1:**
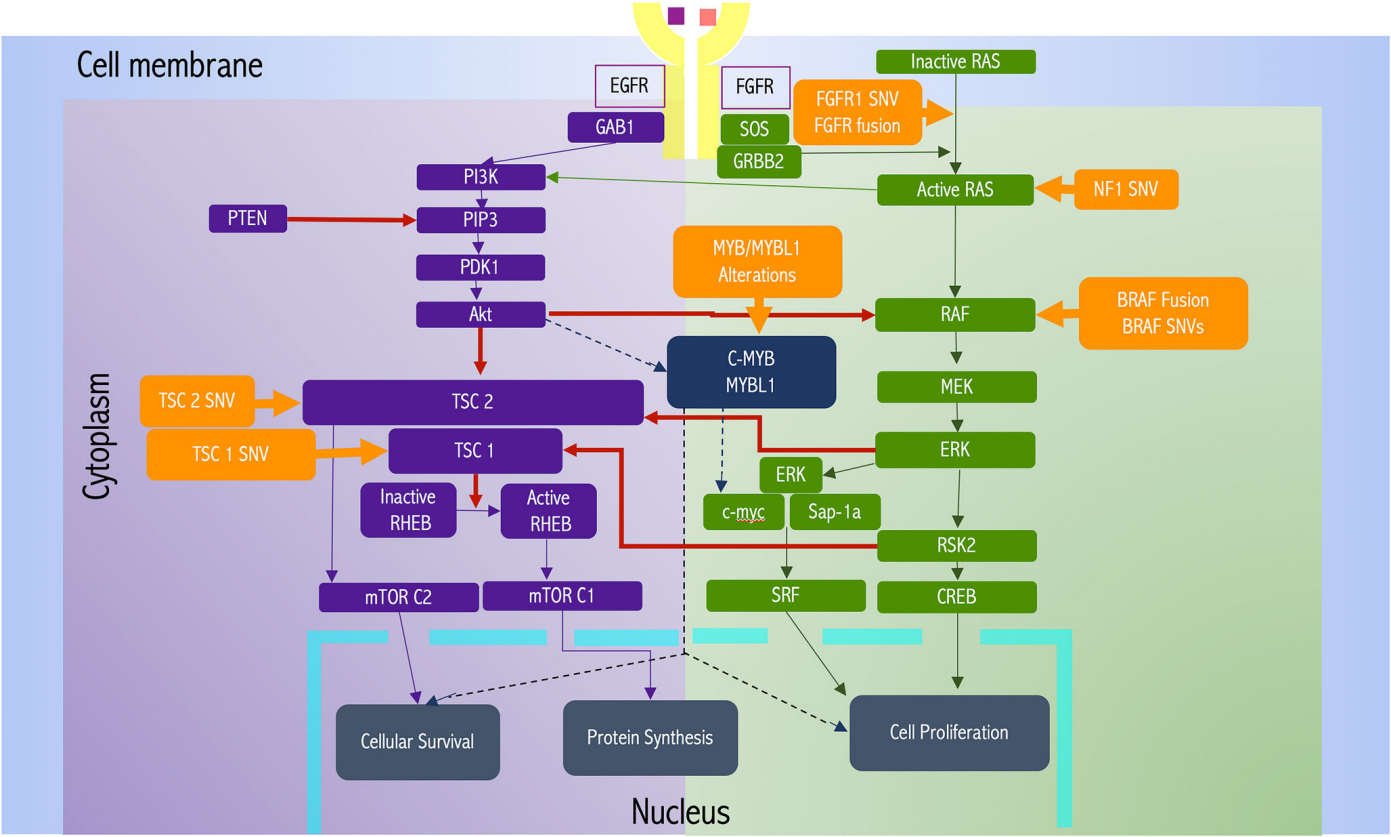
Mitogen-activated protein kinase (MAPK) pathway (green background on the right) and mammalian target of rapamycin (mTOR) pathway (purple background on the left) in tumorigenesis of PLGNTs. Orange boxes show the genetic alterations that drive the tumorigenesis along these pathways. The dashed line indicates weak interaction of the MYB/MYBL1 with the MAPK and mTOR pathway

We now know that almost all of PLGNTs are driven by a single genetic event that activates the mitogen-activated protein kinase (MAPK) pathway (Fig. 1) [8]. Whole-genome sequencing studies showed alterations in the MAPK pathway in almost all pilocytic astrocytomas (PAs), without any other genetic alterations [9]. The evidence of MAPK pathway activation in PLGNTs is so overwhelming that it has been postulated that PLGNTs can be considered one-pathway disease [5, 10].

### MAPK pathway in PLGNT

MAPK is a protein kinase that specifically phosphorylates serine and threonine residues of target proteins. This signaling cascades to transduce a diverse array of stimuli from the cell membrane to the nucleus to maintain cellular homeostasis of gene expression controlling cellular proliferation, differentiation, survival, and apoptosis [11].

Dysregulation of the MAPK pathway leads to tumorigenesis and is common in a wide range of low-grade and malignant CNS tumors [12]. The MAPK pathway can be activated (a) at the level of the cell membrane via overexpression of cell surface receptors by gene amplification, which makes the cell hyperresponsive to normal ambient cell-receptor ligands (e.g., EGFR amplification), and (b) within the cytoplasm via genetic alterations of specific MAPK pathway proteins. These genetic alterations can involve inhibitory mutations in tumor suppressor genes (e.g., *NF1*), gain-of-function mutations in proto-oncogenes (e.g., *BRAF* mutation), or fusion of genes of MAPK pathway proteins with other genes (e.g., *BRAF* fusion) [12].

Activation of the MAPK pathway that leads to tumorigenesis results from two mutually exclusive genetic alterations: (a) genetic rearrangements such as duplication, fusions, or translocations; or (b) single nucleotide variation (SNV) mutations. Although these genetic alterations are not specific to one particular histologic phenotype, clinical outcomes of patients depend on the type of genetic alteration that leads to tumorigenesis. Clinical profiles of patients having genetic rearrangement–driven tumors are significantly different from those having SNV-driven tumors. Regardless of the histologic phenotype, tumors having genetic rearrangements occur in younger children and have a less aggressive clinical course, whereas SNV-driven tumors occur in relatively older children (> 5 years) and are associated with poorer outcomes [8]. Given these observations, pediatric neuro-oncologists prefer to stratify PLGNTs by their genetic alteration patterns [5, 8], although the WHO classification scheme does not yet include genetic information.

#### Major genetic alterations leading to MAPK pathway activation in PLGNTs

##### Genetic rearrangements

KIAA1549-BRAF fusion: The KIAA1549-BRAF fusion is the most frequent molecular alteration in patients with PLGNTs [5, 8, 13]. This fusion result from a ~2-Mb tandem duplication at 7q34 [14]. The most common fusion occurs between the KIAA1549 exon 16 and BRAF exon 9, followed by the 15:9 fusion, 16:11 fusion, 18:10 fusion, and 19:9 fusion, in decreasing order of prevalence [5, 14]. All these genetic alterations code for a fusion protein in which the BRAF kinase domain is retained but the N-terminal regulatory region of *BRAF* is lost, resulting in constitutive activation of the BRAF kinase and downstream upregulation of the MAPK signaling pathway [13, 14].

Histologic significance: KIAA1549-BRAF fusion is characteristically associated with PAs and tumors arising in the posterior fossa, especially the cerebellum [5, 8, 13, 15]. However, KIAA1549-BRAF fusion has been found at other CNS locations and in other histologic phenotypes [5].

Imaging significance: Tumors harboring the (16:9) KIAA1549-BRAF fusion tend to arise from midline structures and have a predilection for infratentorial structures, especially the cerebellum [5, 13]. Additionally, optic pathway tumors are commonly driven by (16:9) KIAA1549-BRAF fusion. PLGNTs with (15:9) KIAA1549-BRAF fusion more commonly arise from the supratentorial compartment or at the midline [16]. Tumors with (16:9) KIAA1549-BRAF fusion are usually well-circumscribed, whereas tumors with (15:9) KIAA1549-BRAF fusion tend to extensively disseminate [8].

Prognostic significance: Patients having tumors with the (16:9) KIAA1549-BRAF fusion have excellent prognosis, with 5-year progression-free survival (PFS) rates of 77–100% and rare progression or recurrence [8]. This excellent prognosis might be due to tumors with this genetic fusion being well-circumscribed and typically affecting the cerebellum, which is easily amenable to gross total resection [5, 17]. Patients with PLGNTs with (15:9) KIAA1549–BRAF fusion, however, have a poorer prognosis with a 5-year PFS rate of only 59% [8].

FGFR alterations: The fibroblast growth factor receptor (FGFR) family consists of four highly conserved transmembrane tyrosine kinase receptors (FGFR1–4) and represents a receptor tyrosine kinase (RTK) signaling pathway that transduces signal from the cell surface to the nucleus by activating the intramembranous tyrosine kinase domain [18]. Two major patterns of *FGFR1* gene arrangement are common in PLGNTs: FGFR1 tyrosine kinase domain duplication (FGFR1-TKDD) and FGFR1-transforming acidic coiled-coil protein 1 fusions (FGFR1-TACC1). A functional study showed that FGFR1-TKDD induces FGFR1 autophosphorylation and upregulation of both MAPK/ERK and PI3K pathways. FGFR1-TACC1 fusions also constitutively activate FGFR and cause downstream activation of the MAPK pathway [19]. FGFR2–Catenin Alpha 3 fusion (FGFR2-CTNNA3) is another more recently described FGFR alteration that is thought to result in homodimerization and autophosphorylation of FGFR2 and downstream MAPK [20].

Histologic significance: Histologic manifestations of FGFR alterations are variable. FGFR1 alterations are enriched in PLGNTs with an oligodendroglial phenotype [21]. The FGFR1-TACC1 fusion is typically associated with extra-ventricular neurocytomas having an oligodendroglioma-like appearance on histology [22]. FGFR1-TKDD is characteristically seen in dysembryoplastic neuroepithelial tumors (DNTs), which also demonstrate oligodendroglial morphology on histology. FGFR1-TKDD is also associated with pediatric-type diffuse low-grade gliomas (PDLGGs) with oligodendroglial phenotype on histology [23]. FGFR2-CTNNA3 fusion is characteristically found in polymorphous low-grade neuroepithelial tumor of the young (PLNTY), another tumor with oligodendroglial morphology on histology [8]. However, FGFR1 alterations have also been described in tumors with astrocytic lineage. Both FGFR1-TACC1 fusion and FGFR1-TKDD fusion have been described in extracerebellar PAs [5, 23].

**Fig. 2:**
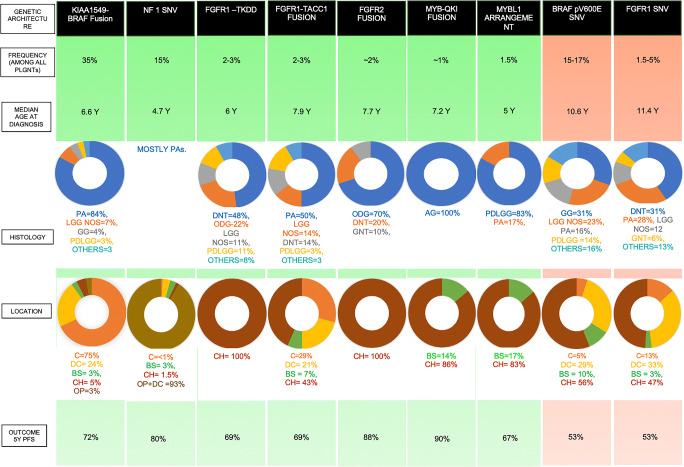
Characteristics of specific genetic alterations. Based on the genetic profile, location, age at presentation, and histology, the PLGNT phenotypes can be stratified as (a) low risk, requiring a conservative approach involving surgical resection and wait and watch; (b) intermediate risk, necessitating proactive engagement with surgical resection, chemotherapy with or without targeted therapy, and close surveillance; and (c) high risk, requiring aggressive management with surgical resection, chemotherapy, and considerations for clinical trials. [5] Green background indicates the low risk profile associated with these genetic rearrangements and SNVs. Orange background suggests intermediate risk associated with these genetic SNVs. Data from Ryall et al [5]. Abbreviations: PA, pilocytic astrocytoma; PDLGG, pediatric diffuse low-grade glioma; PLGNT, pediatric low-grade neuroepithelial tumors, GG, ganglioglioma; DNT, dysembryoplastic neuroepithelial tumor; ODG, oligodendroglioma; LGG NOS, low-grade glioma not otherwise specified; RGNT, rosette-forming glioneuronal tumor; BS, brainstem; C, cerebellum; CH, cerebral hemispheres; DC, diencephalic; OP, optic pathway

Imaging significance: All tumors associated with FGFR1/2 alterations preferentially affect the cerebral hemispheres [8]. Tumors associated with FGFR1-TKDD (including PAs) and FGFR2 fusions preferentially affect cortical/juxtacortical regions of the cerebral hemisphere [23]. Tumors associated with FGFR1-TACC1 fusion commonly have cystic components (Fig. 2) [8].

Prognostic significance: Patients with tumors associated with FGFR1/2 alterations have an excellent overall survival (OS) rate. The 5-year PFS rate varies from 69% in those with FGFR1-TACC1 and FGFR1-TKD fusions to 88% in those having the FGFR2 fusion [8].

MYB/MYBL1 alterations: The MYB proto-oncogene protein (c-MYB) plays an important role in controlling proliferation and differentiation of hematopoietic and other progenitor cells. The MYB-QKI fusion is a major MYB alteration, enriched in angiocentric gliomas (AGs) [24]. Rarely, the proto-oncogene *MYBL1* (MYB Like 1) is involved in the development of PLGNTs.

Histologic significance: Unlike tumors with FGFR alterations, MYB alterations are typically associated with astrocytic tumors [5, 21]. MYB-QKI fusion is found in 87% of AGs and 41% of pediatric diffuse low-grade gliomas (PDLGGs) (Fig. 2). MYBL1 alterations are associated with diffuse isomorphic glioma.

Imaging significance: Most PLGNTs with MYB/MYBL1 alterations are hemispheric tumors; the brain stem and diencephalon are involved less commonly [5, 24].

Prognostic significance: Tumors with MYB and MYBL1 alterations occur in young children, and their prognosis is excellent, with a 10-year OS rate of 90% [24].

##### Single nucleotide variation

BRAF: The BRAF p.V600E mutation, in which single valine nucleotide is replaced with glutamic acid at position 600, constitutively activates the RAS/MAPK pathway by acting as a phosphomimetic. This BRAF p.V600E SNV is the second most common genetic alteration in PLGNTs. Like other SNV-driven tumors, those with the BRAF p.V600E SNV are frequently associated with additional SNVs, most commonly NF1, FGFR1, KRAS, and H3F3A [8].

Histologic significance: Unlike PLGNTs with KIAA1549-BRAF fusions, tumors with BRAF p.V600E SNVs are histologically diverse, with this mutation occurring in up to 80% of pleomorphic xanthoastrocytoma (PXAs), 40% of PDLGGs, and 45% of GGs (Fig. 5) [25]. It is less common in PAs and glioneuronal tumors. A rare variant of the BRAF SNV, BRAF p.V600D, has also been described in desmoplastic infantile ganglioglioma (DIG) [26].

Imaging significance: Compared to PLGNTs with KIAA1549-BRAF fusions, which are spatially enriched in the cerebellum and midline structures, tumors with BRAF p.V600E SNVs are more common in the supratentorial compartment, occurring most frequently in cerebral hemispheres. Involvement of midline structures such as the diencephalon and brainstem is not uncommon, but cerebellar involvement is rare [27, 28].

Prognostic significance: Patients having PLGNTs with BRAF p.V600E mutation have poorer OS and PFS rates than BRAF fusions do [27]. Because tumors having BRAF p.V600E mutation frequently involve deep brain structures, complete resection is rarely achieved. Furthermore, BRAF p.V600E mutation is frequently associated with additional SNVs, including homozygous CDKN2A deletions, that carry the risk of transforming a PLGNT to a higher-grade glioma, specifically in PXAs [29]. Both incomplete resection and additional mutations especially CDKN2A deletions are independent predictors of poor outcome [27]. Targeted therapy using BRAF inhibitors (i.e., dabrafenib) is an excellent treatment option in tumors with BRAF pV600E SNV with a robust and durable responses [30].

Neurofibromatosis 1 (NF1): NF1 is caused by a germline mutation in the *NF1* tumor suppressor gene that functions as a negative regulator of the RAS/MAPK pathway (Fig. 1). PLGNTs associated with NF1 result from loss of the wild-type allele, resulting in activation of the RAS/MAPK pathway due to loss of function of neurofibromin, a tumor suppressor protein. NF1-associated low-grade gliomas, usually seen at younger ages, have very low mutation burden, with only a few somatic mutations [31]. In contrast, higher-grade gliomas, which are more common in adults with NF1, have a higher mutation burden involving ATRX, TP53, and CDKN2A [31].

Histologic significance: In up to 15% of patients with NF1, PLGNTs develop in the optic pathway; in another 5%, PLGNTs develop in other regions of the CNS (Fig. 2).

Imaging significance: In children, tumors with NF1 mutation are typically midline PAs with an unusual propensity to involve the optic pathway. The brainstem is the second most common site of tumor with NF1 mutation.

Prognostic significance: Optic pathway PLGNTs in patients with NF1 are mostly asymptomatic and indolent and do not require treatment. In some cases, the tumor regresses completely over time without any treatment [13]. Some optic pathway tumors in younger children (<5 years old) can have concomitant involvement of the medial temporal lobes, basal ganglia, and thalamus [32]. These “deep extensive tumors” are associated with nonvisual CNS symptoms and confer a worse prognosis, with a PFS rate of only 4 years [32]. PLGNT associated with NF1 can develop outside the optic pathway in up to 19% of patients [8]. Patients with non-optic pathway tumors have poorer prognosis than do patients with optic pathway tumors, particularly if the children are younger (<2 years old) and the tumors have additional mutations [8]. Up to 63% of non-optic pathway gliomas progress over time [33]. Historically, patients with PLGNTs with NF1 mutation were either not or rarely biopsied because they have favorable outcomes. However, given the positive correlation between additional genetic alterations and resultant poorer outcomes in patients with non-optic pathway NF1 tumors, a biopsy is justified to identify at-risk patients early in the disease’s course of management [5, 8].

FGFR1: Two hot spot mutations (p.N546K and p.K656E.) in the tyrosine kinase domain of FGFR1 can constitutively activate FGFR1 and activate the MPAK pathway. These hot spot mutations occur in up to 10% of PLGNTs.

Histologic significance: An FGFR1 SNV is seen in 1.8% of PLGNTs, most frequently in DNTs and extracerebellar PAs [8], and in PDLGGs [7]. FGFR1 SNVs have also emerged as the molecular hallmark of rosette-forming glioneuronal tumor (RGNT) [34].

Imaging significance: Like other FGFR1 alterations, most tumors associated with the FGFR1 SNV preferentially affect cortical/juxtacortical regions of the cerebral hemisphere.

Prognostic significance: Tumors with FGFR1 SNV progress rapidly (median progression time is 2.2 years) with a 5-year PFS rate of only 53% [8].

#### Secondary genetic alterations in PLGNTs

CDKN2A deletion: The homozygous deletion of 9p21.3 results in loss of the tumor suppressor CDKN2A, which regulates the G1cell cycle. In contrast to adult low-grade gliomas, PLGNTs do not commonly lose CDKN2A expression, with an incidence of loss of 6–20% in PLGNTs. Within the PLGNT family, this mutation is a common secondary mutation in tumors with the BRAF V600E SNV, specifically in PXAs [5, 35]. A combination of BRAF V600E SNV and CDKN2A deletion has also been described in PDLGGs and in hemispheric PAs [35]. PLGNTs with both BRAF p.V600E and CDKN2A deletions represent a distinct clinical subtype that frequently shows more aggressive histologic behavior and is prone to transformation into secondary HGG [29]. Accordingly, tumor progression or recurrence may be encountered more frequently during follow-up imaging of these PLGNTs.

## Epigenetic landscape of PLGNTs

The interplay of epigenetic and genetic alterations in PLGNTs is not fully understood. However, epigenetic analysis using DNA methylation arrays has already improved our understanding of and ability to diagnose and risk-stratify pediatric brain tumors [6]. Epigenomic analysis has uncovered new molecular entities and allowed more accurate classification and subclassification of tumors, which has helped identify prognostic risk groups and may allow the development of epigenetics-based tailored therapy [6, 36–38]. Epigenetics-based subclassification of PLGNTs suggests that a specific DNA methylation status is more tightly linked to tumor location than genetic alterations are [6]. Midline optic pathway and cerebellar tumors are clustered together. Even for the same histologic subtypes with similar genetic alteration, the DNA methylation profile of diencephalic tumors differs from that of hemispheric tumors [6].

The epigenetic landscape of PLGNTs is rapidly changing. Currently, epigenetics is being sparingly used to characterize specific PLGNTs, but in the near future, epigenetic information will likely be extensively used to characterize PLGNTs.

## Histologic landscape of PLGNTs

PLGNTs are an extremely heterogeneous group of neoplasms arising predominantly from the glial cell lineage that includes astrocytic, oligodendrocytic, or mixed neuronal and glial lineage. These tumors are classified as WHO grade I and II tumors [3]. Although PLGNTs encompass numerous histologic phenotypes, they can manifest either as a focal well-circumscribed tumor or as a diffuse infiltrating tumor with ill-defined margins.

### Focal well-circumscribed phenotypes of PLGNTs

PAs are the most common well-circumscribed PLGNTs (up to 85%). Although PAs can arise anywhere along the neuraxis, most involve the cerebellum and midline structures such as the brainstem and the optic pathway [39]. Most of the other well-circumscribed tumors involve the cerebral hemispheres and constitute up to 20% of all biopsied PLGNTs [40, 41]. The incidence of cerebral hemispheric PLGNTs in order of decreasing frequency is as follows: gangliogliomas (GGs; 23%), dysembryoplastic neuroepithelial tumors (DNTs; 18%), PLGNTs not otherwise specified (12%), glioneuronal tumors (9%), oligodendrogliomas (6%), pleomorphic xanthoastrocytoma (PXAs; 5%), and subependymal giant cell astrocytoma (SEGA; 4%) [4].

### Diffuse phenotypes of PLGNTs

Unlike the well-circumscribed tumors, the diffuse low-grade gliomas with infiltrative margins are uncommon in the pediatric population [7], with an incidence of 8% [4]. Pediatric-type diffuse low-grade gliomas (PDLGGs) are enriched in BRAF p.V600E mutation, FGFR alteration, or MYB or MYBL1 rearrangement [7]. This phenotype has an indolent clinical course with rare anaplastic progression [7]. Patients with this phenotype generally have a prolonged disease course and good OS, despite experiencing significant morbidity during their chronic disease [7]. PDLGGs have no genetic hallmark. BRAF p.V600E mutation, FGFR alterations, and MYB/MYBL1 rearrangement are common in PDLGGs and these genetic alterations significantly influence prognosis of PDLGGs; it has been suggested to classify PDLGGs on the basis of these specific genetic alterations [7].

Although there are radiological and histological similarities, PDLGG and diffuse low-grade gliomas in adults have clinically significant differences: Adult diffuse low-grade gliomas are most commonly IDH-mutated astrocytoma or oligodendroglioma with concomitant 1p/19q deletion. These adult-type tumors have a more aggressive clinical course and higher chances of transformation into a higher-grade glioma. IDH wild-type and histone H3 wild-type PDLGGs are indolent tumors with low risk of malignant transformation.

Similar to PDLGGs, polymorphous low-grade neuroepithelial tumor of the young (PLNTY) has infiltrating margins. PLNTYs are usually smaller tumors that typically present with seizures due to preferential involvement of the cortical/juxtacortical regions.

Diffuse leptomeningeal glioneuronal tumor (DLGNT) is another diffuse tumor that specifically involves the leptomeningeal surface of the spinal cord and posterior fossa. It is also biologically distinct from PDLGGs as this tumor frequently harbors BRAF fusions and 1p deletions [33].

## Clinical landscape of PLGNTs

The clinical presentation of PLGNTs largely depends upon the location of the tumor. Glioneuronal and neuronal phenotypes of PLGNTs usually arise from superficial aspects of the brain (cortical/juxtacortical regions); they typically present with a long history of epilepsy and are commonly known as long-term epilepsy-associated tumors (LEATs) [42]. BRAF p.V600E SNV, FGFR1 alterations, and MYB alterations are the common drivers of LEATs. NF1-associated tumors preferentially arise from the optic pathway and present with altered vision. Tumors with KIAA1549-BRAF fusion commonly involve the cerebellum and present with gait or coordination problems, with or without features of increased intracranial pressure. Tumors around the foramen of Monro, such as SEGA (due to TSC1 and TSC2 SNV) and septal dysembryoplastic neuroepithelial tumors (sDNT), frequently result in obstructive hydrocephalus. Tectal glioma (TG) and larger posterior fossa tumors also can produce hydrocephalus by compressing the aqueduct of Sylvius and the 4th ventricle respectively. Duration of symptoms also depends upon the location of the tumor. A tumor closer to eloquent brain areas presents early, whereas a tumor arising from non-eloquent areas presents relatively late. As a result, a tumor arising from the non-eloquent brain areas, regardless of its histologic phenotypes, is usually larger at presentation.

Although any PLGNT phenotype can present anytime during the childhood, some phenotypes preferentially involve a specific age group. For example, desmoplastic infantile astrocytoma and desmoplastic infantile ganglioglioma (DIA and DIG) present very early (<2 years), whereas PLNTY typically affects older teens (median age of presentation, 17.5 years). PA, the most common PLGNT phenotype, most commonly occurs before 20 years of age.

## Imaging landscape of PLGNTs

The imaging landscape of PLGNTs is tightly linked to tumor location and histology. Well-circumscribed, focal tumors tend to appear well-circumscribed on imaging [43]. Similarly, PDLGGs are ill-defined both in histology and on imaging. The imaging appearance of the internal morphology of a specific PLGNT phenotype also depends on the specific histologic phenotype (cellular density, incidence of mitotic figures, type of matrix, vascularity, etc.) Tumors with hypercellularity, such as DIA and DIG and some of the PXAs, demonstrate diffusion restriction, whereas tumors with loose matrix, such as DNTs, demonstrate no diffusion restriction. The cystic component of the tumors demonstrates facilitated diffusion. The perfusion characteristics of a specific PLGNT phenotype depend upon histology. Unlike in high-grade gliomas, neo-angiogenesis is not a dominant histologic feature of PLGNTs. Expectedly, low perfusion is the typical appearance of PLGNTs on perfusion imaging. Tumors with leaky blood vessels demonstrate enhancement. Elevated choline and low N-acetyl aspartate (NAA) are the most common findings on MR spectroscopy (MRS). Elevated lactate has been described in PAs and PGNTs. Elevated mI has been described in DNTs and in AGs.

**Fig. 3:**
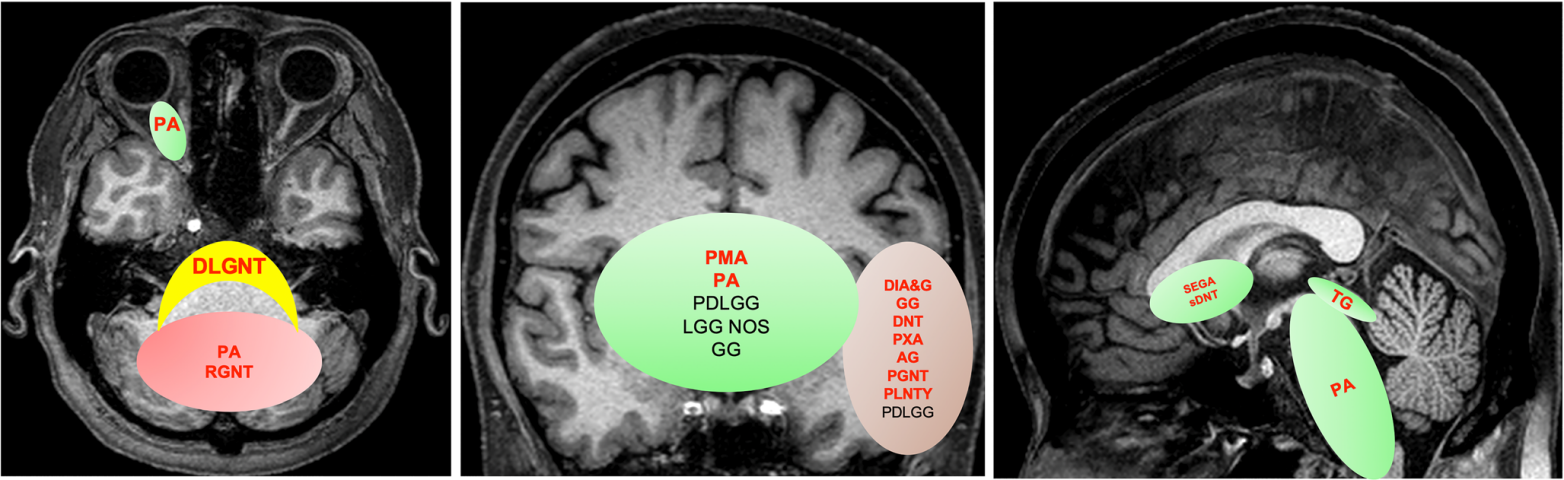
Spatial clustering of PLGNT phenotypes. Red color represents cerebellar clustering; green color represents midline tumors, and brown represents hemispheric tumors that include both cortical/juxtacortical tumors and deep brain tumors. Red font indicates the preferred site for that individual PLGNT phenotype. Abbreviations: AG, angiocentric glioma; DIA&G, desmoplastic infantile astrocytoma and ganglioma; DLGNT, diffuse leptomeningeal glioneuronal tumor; DNT, dysembryoplastic neuroepithelial tumor; GG, ganglioglioma; IDG, isomorphic diffuse glioma; LGG NOS, low-grade glioma not otherwise specified; PA, pilocytic astrocytoma; PDLGG, pediatric diffuse low-grade glioma; PGNT, papillary glioneuronal tumor; PLGNTs, pediatric low-grade neuroepithelial tumor; PLNTY, polymorphous low-grade neuroepithelial tumor of the young; PMA, pilomyxoid astrocytoma; PXA, pleomorphic xanthoastrocytoma; RGNT, rosette-forming glioneuronal tumor; SEGA, subependymal giant cell astrocytoma; sDNT, septal DNT; TG, tectal glioma

**Fig. 4:**
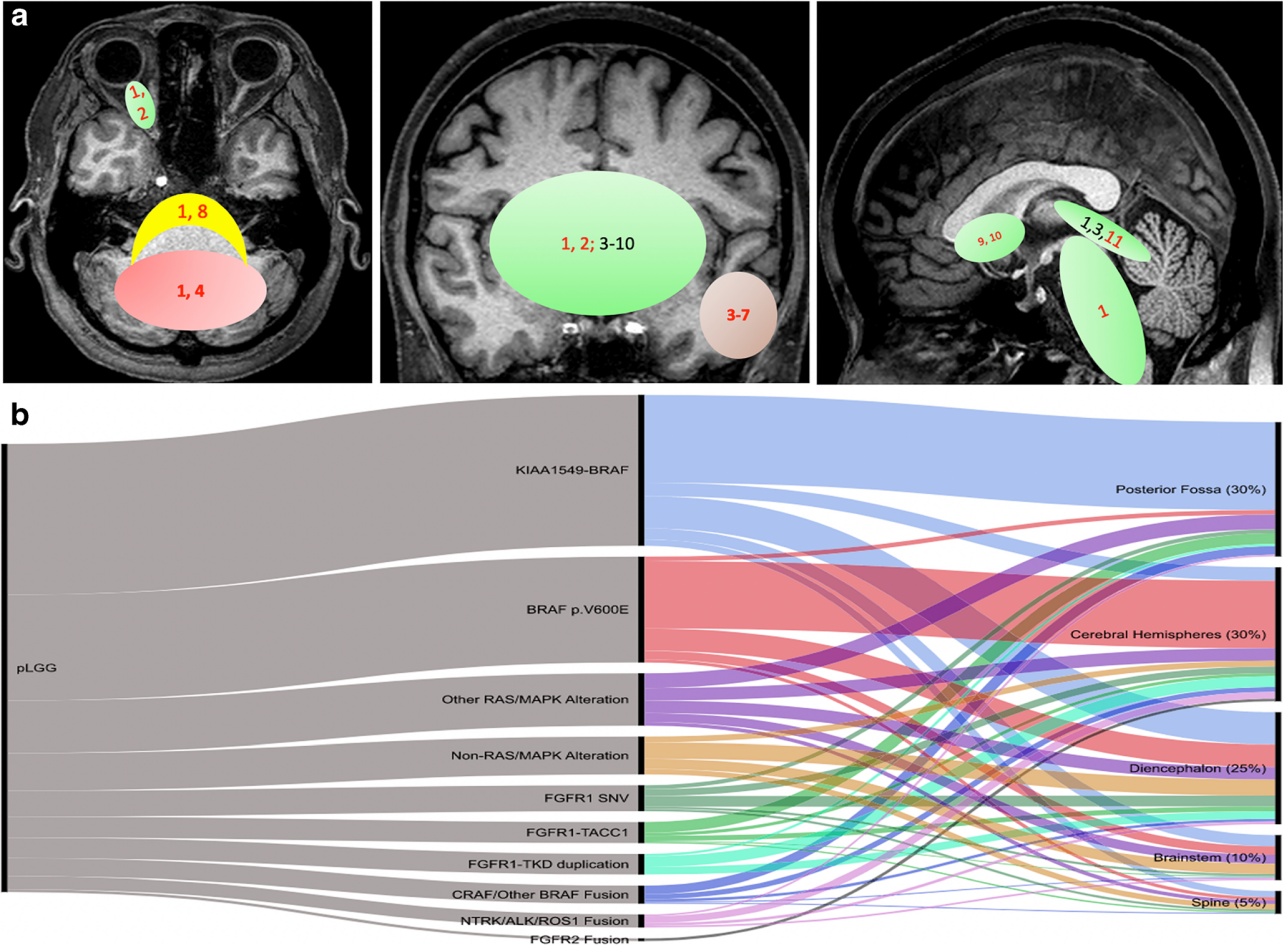
Spatial enrichment of specific genetic mutations. **a** Spatial clustering of PLGNT phenotypes. Red color represents cerebellar clustering; green color represents midline tumors, and brown represents hemispheric tumors that include both cortical/juxtacortical tumors and deep brain tumors. Red font indicates the preferred site for that individual PLGG phenotype. 1=KIAA1549-BRAF fusion; 2=NF1 SNV; 3=BRAF p.V600E SNVs; 4=FGFR1 SNV, 5=MYB/MYBL1 arrangements; 6=PRKCA arrangement; 7=BRAF p.V600D/E. arrangement; 8=1p del and --KIAA1549-BRAF fusion; 9=TSC 1&2 SNV; 10=PDGRFA SNV; 11= KRAS G12R SNV. **b** Distribution of molecular alteration and location as per Ryall et al [5]

Spatial enrichment of individual PLGNT phenotype ultimately depends upon the underlying genetic changes leading to tumorigenesis, which are important for both histology and imaging. The differential diagnoses of a brain tumor is largely dependent upon the epicenter of a tumor. Spatial enrichment of genetic changes and histology are described in Figs. 3 and 4.

## Radiohistogenomic stratifications of PLGNTs

**Fig. 5:**
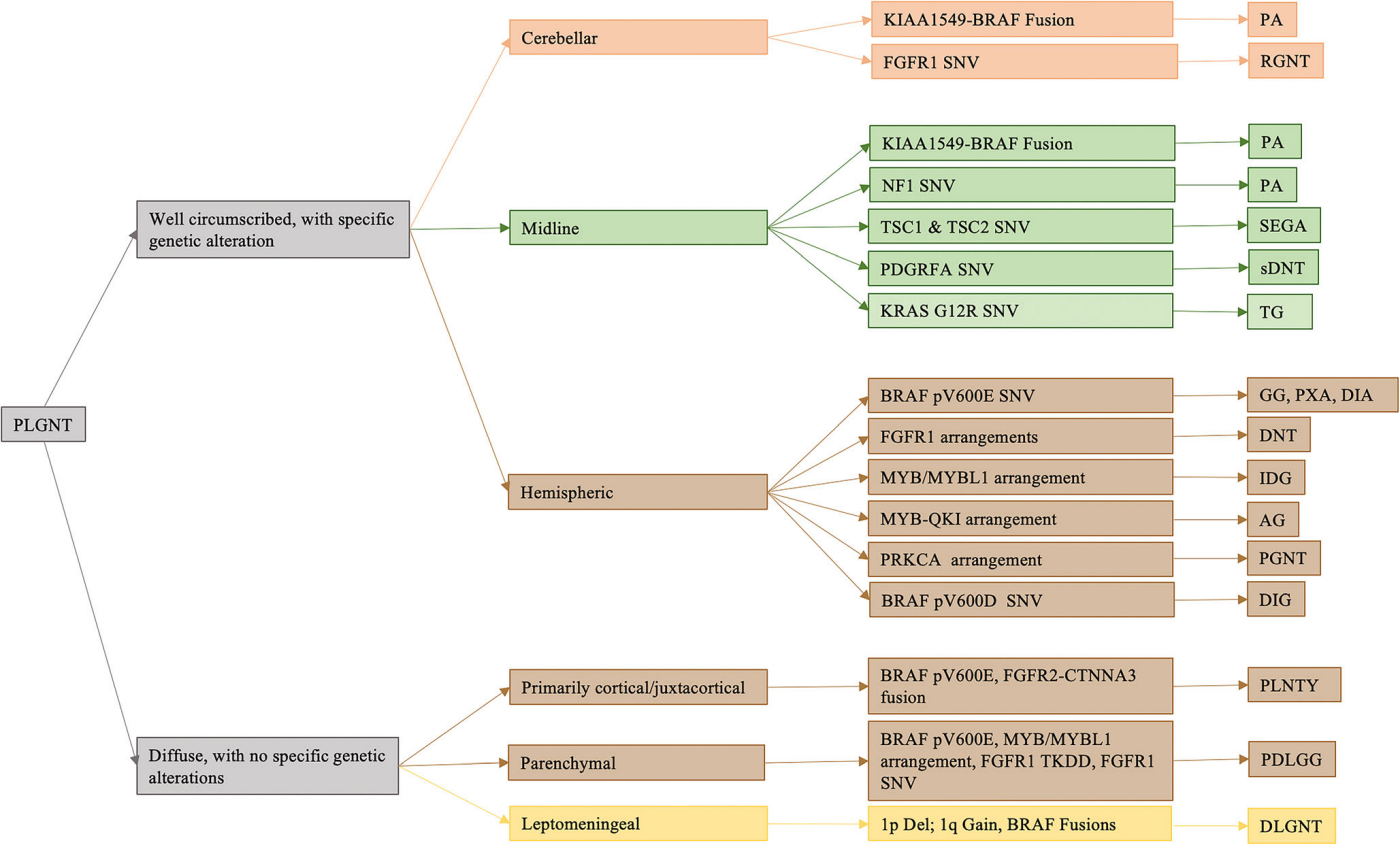
Imaging stratifications of PLGNTs by tumor margin, location, and genetic alteration. Specific genetic alteration is defined as the frequency of a characteristic mutation in >50% of tumors. Abbreviations: AG, angiocentric glioma; DIA, desmoplastic infantile astrocytoma; DIG, desmoplastic infantile ganglioma; DLGNT, diffuse leptomeningeal glioneuronal tumor; DNT, dysembryoplastic neuroepithelial tumor; GG, ganglioglioma; IDG, isomorphic diffuse glioma; LGG NOS, low-grade glioma not otherwise specified; PA, pilocytic astrocytoma; PDLGG, pediatric diffuse low-grade glioma; PGNT, papillary glioneuronal tumor; PLGNT, pediatric low-grade neuroepithelial tumor; PLNTY, polymorphous low-grade neuroepithelial tumor of the young; PMA, pilomyxoid astrocytoma; PXA, pleomorphic xanthoastrocytoma; RGNT, rosette-forming glioneuronal tumor; SEGA, subependymal giant-cell astrocytoma; sDNT, septal dysembryoplastic neuroepithelial tumor; TG, tectal glioma

Tumor margin and location are the two key imaging parameters that are universally used by radiologists to generate meaningful image-based differential diagnoses. Most PLGNTs are well-circumscribed, so we primarily classify PLGNTs as either well-circumscribed or diffuse. Because well-circumscribed PLGNT phenotypes are driven by specific genetic alterations and are spatially enriched, we sub-stratify the well-circumscribed PLGNTs based on their preferred CNS location. We use a three-tier system to classify individual histologic phenotypes of the PLGNT family on the basis of tumor margin, tumor location, and the presence or absence of a characteristic genetic alteration. Tumor margin is defined as well-circumscribed or diffuse. Tumor location is defined as (a) cerebellar, (b) involving midline structures (the midline is defined by median and paramedian brain structures in both the supratentorial and infratentorial compartments and includes the optic pathway, diencephalon, brain stem, and basal ganglia), (c) hemispheric, or (d) leptomeningeal (Fig. 5)

This three-tier approach to radiohistogenomic stratification of PLGNTs is not 100% diagnostic and has limitations. A major shortcoming is that each PLGNT phenotype can involve any CNS location, although it might have a preferred site. For example, PA characteristically arises from cerebellum, but it can also arise from cerebral hemispheres on rare occasions. As imaging appearances of internal tumor morphology (e.g., cyst with a mural nodule) are often linked to specific histologic phenotypes, correct identification of each phenotype is still feasible even if it arises from its non-preferred sites. However, radiologic diagnosis of a specific PLGNT phenotype becomes a challenge if it arises from a non-preferred site and lacks characteristic imaging findings.

### Focal well-circumscribed PLGNTs with characteristic genetic alterations

#### PLGNTs arising from cerebellum

##### Tumors with KIAA1549-BRAF fusion

**Pilocytic astrocytoma (PA)**

PAs can arise anywhere in the CNS; in children, they most frequently occur in the infratentorial compartment, with the cerebellum being the most commonly involved structure [13, 39]. Other commonly involved areas include midline brain structures such as the optic pathway, diencephalic structures, brain stem, and spinal cord [39]. Although the histology of PAs in different locations within the neuraxis is mainly the same, a few site-specific variations exist [44].

Histology: PAs are characteristically well-circumscribed tumors with low to moderate cellularity and consist of compacted bipolar cells with Rosenthal fibers and loosely textured multipolar cells in a mucoid background with microcyst formation [13]. Mitoses and hyperchromatic and pleomorphic nuclei are rare but compatible with the diagnosis of PA [13].

Microvascular proliferation leading to thick-walled vessels that are either hyalinized, glomeruloid, or both is frequently seen in the solid component of PAs and along the wall of the cyst. Microvascular proliferation in PAs differs significantly from that in glioblastoma in terms of structural and genetic expression [45]. Neoangiogenic vessels in PA and glioblastoma are leaky, which explains the contrast enhancement in both tumor types, but neoangiogenic vessels are wider and less dense in PA than in glioblastomas [46]. The three-layered structure of neoangiogenic vessels is more mature and less dense in PAs than in glioblastomas [45]. Neoangiogenic vessels in PAs are inefficient, leading to infarct-like necrosis without pseudopalisading, a feature that is common in PAs [13].

Some cerebellar PAs have a diffuse growth pattern, which might be the most dominant histologic finding. Patterns of microvascular proliferation of cerebellar PAs can also vary from one location to another. For example, cerebellar PAs have a wider caliber of vessels than do other sites [46].

Up to 82% of cerebellar PAs have the KIAA1549-BRAF fusion [47]. Other RAF fusions and BRAF pV600E mutations have been described in PAs but are less common in cerebellar PAs.

**Fig. 6:**
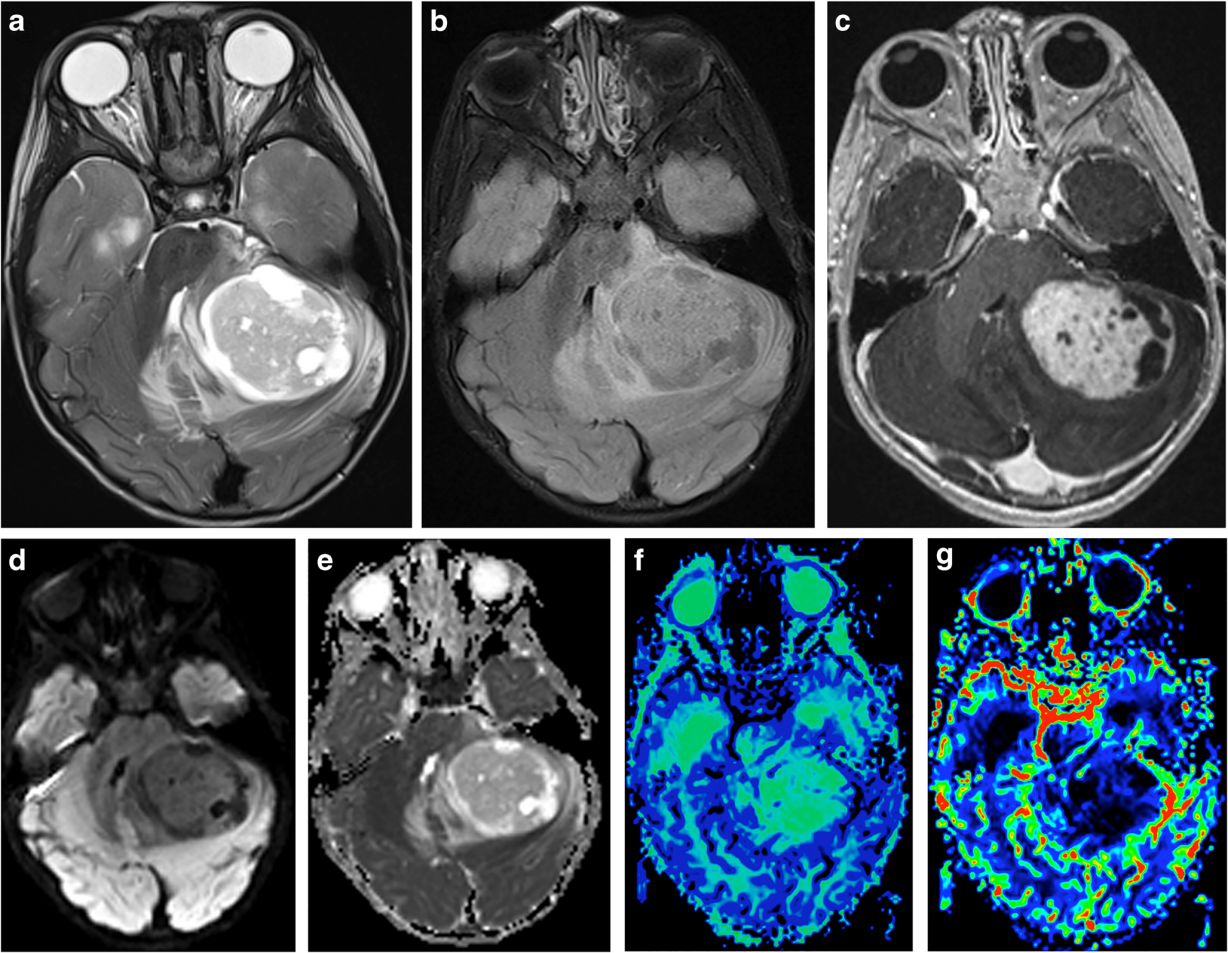
Pilocytic astrocytoma. **a** Axial T2-weighted scan through the cerebellum demonstrating a well-defined solid-cystic mass in the left cerebellar hemisphere that is heterogeneous onT2-weighted sequence and causes mass effect to the 4th ventricle. The solid component is minimally hyperintense compared to the gray matter. **b** An axial T2 FLAIR image through the same level better demonstrates the area of T2 hyperintensity beyond the tumor margin, suggestive of edema. **c** An axial post-contrast T1-weighted sequence through the same level demonstrates intense enhancement of the solid component of the tumor. **d** The axial diffusion image and ADC map (**e**) through the same level show no diffusion restriction. **f** The mean transit time map demonstrates increased transit time within the solid component of the tumor. **g** The cerebral blood volume map shows that the tumor has very low cerebral blood volume

Imaging: The characteristic imaging finding in cerebellar PA is a cystic tumor with a mural nodule. Due to loose matrix and/or the presence of microcysts as described above, the solid mural nodule is usually isodense on CT and hyperintense compared to the gray matter on T2-weighted sequences [48]. For the same reason, ADC of the solid component is typically >1400 × 10^–6^ mm^2^s^–1^, in contrast to <800 × 10^−6^ mm^2^s^−1^ in medulloblastomas [49]. The nodule typically demonstrates intense enhancement on the post-contrast T1-weighted sequence due to leaky tumoral vessels (Fig. 6). Enhancement of the cyst wall is variable. When present, it could be due to either tumor enhancement or reactive enhancement caused by intra-cystic hemorrhage, which is not uncommon in PAs.

The literature on perfusion parameters of PAs is extremely variable. Some studies show low CBV within the solid component of the PAs [50–52], whereas others show high CBV [53, 54]. Multiple reasons account for this variance: (a) variability in the perfusion imaging acquisition techniques, (b) use of different algorithms to postprocess the raw perfusion images, and (c) excessive leakiness of the tumor vascularity. The results of most of the studies with conventional T2*-weighted perfusion imaging demonstrate lower CBV of the solid component of the PAs than in high-grade gliomas [50–52]. CBV measured by T1-weighted perfusion imaging, which is not commonly used in clinical practice, is reportedly higher in PAs than in high-grade gliomas [54]. A study using a simple postprocessing algorithm to process the T2*-weighted raw perfusion images has also reported high CBV in PAs [53]. The authors strongly believe that these reported variabilities of the perfusion imaging data are due to technical differences.

On T2*-weighted perfusion images, signal intensity on the signal intensity-time curve of the solid component of the PAs characteristically increases after the nadir and crosses the baseline after the first pass due to prominent T1-shortening effects of the gadolinium-based contrast within the extravascular extracellular space due to excessive leakiness of the neoangiogenic vessels [51–53, 55]. Because of this, the calculated CBV largely depends upon whether or not the postprocessing algorithm includes leakage correction, gamma variate fit, and arterial input function, and on the type of integration of the signal intensity-time curve used by the software, allowing only the first pass versus the more common trapezoidal integration that begins immediately after the baseline imaging and continuing overall acquired time points [56–58]. The currently available postprocessing software programs for clinical use utilize one or a combination of the abovementioned variables in the algorithm. We recommend using consistent methodology, preferably using standardized techniques [56, 57], and to be familiar with the software being used to calculate the CBV map. In our experience, the solid component of the PAs typically shows lower CBV than do adjacent brain tissues if standardized techniques are followed. Time parameters obtained from the perfusion MRI of the solid component can have more diagnostic value than CBV in PAs [59]. Mean transit time (MTT) (Fig. 6) and time to peak are both high in PAs, which suggests that the transfer time through hyalinized narrow tumor blood vessels during the first pass is longer [59], which also explains the inefficiency of the neoangiogenic vascularity in PAs that leads to the propensity to develop infarct-like necrosis on histology. Arterial spin labelling (ASL) perfusion MRI is performed without any contrast administration for quantitative estimation of cerebral blood flow (CBF). CBF in PAs calculated by using the ASL technique significantly correlates with the CBV and CBF calculated by the T2*-weighted perfusion MRI [60]. PAs’ tumor vessel permeability does not significantly differ from that of high-grade gliomas as measured with K^trans^ using dynamic contrast-enhanced T1-weighted perfusion imaging [54].

The nodule typically demonstrates a very high choline peak on MRS, with an increased choline-to-NAA ratio (ranging from 1.8 to 3.1) and choline-to-creatine ratio [61, 62]. An elevated lactate peak is also commonly seen on PAs [62, 63].

Uncommon appearances include a completely solid tumor or multiple cystic conglomerations, and predominantly solid tumors with cystic components [48]. PAs can also be infiltrative and have an ill-defined border or can have an exophytic component in the cerebellopontine angle cisterns [48]. Gross total resection of this tumor can be difficult. Rarely, the tumor can present as a ring-enhancing lesion with central necrosis, similar to glioblastoma.

##### Tumors with FGFR1 SNV

**Rosette-forming glioneuronal tumor (RGNT)**

RGNT is a slow-growing tumor arising from the midline, predominantly from the fourth ventricle. The tumor can also arise from the pineal region.

Histology: RGNT is a typically slow-growing, well-circumscribed tumor composed of a neurocytic component and a glial component. The neurocytic component forms a rosette or perivascular pseudorosette. The neurocytic rosette lies in a loose microcystic mucinous matrix. The glial component dominates the tumor. Invasion into the adjacent vermis is common. The glial component could be microcystic or fibrillary.

FGFR1 SNV is the genetic hallmark, which is present in all RGNTs [64]. Concomitant presence of a PIK3CA SNV can be seen in up to 63% [64].

**Fig. 7:**
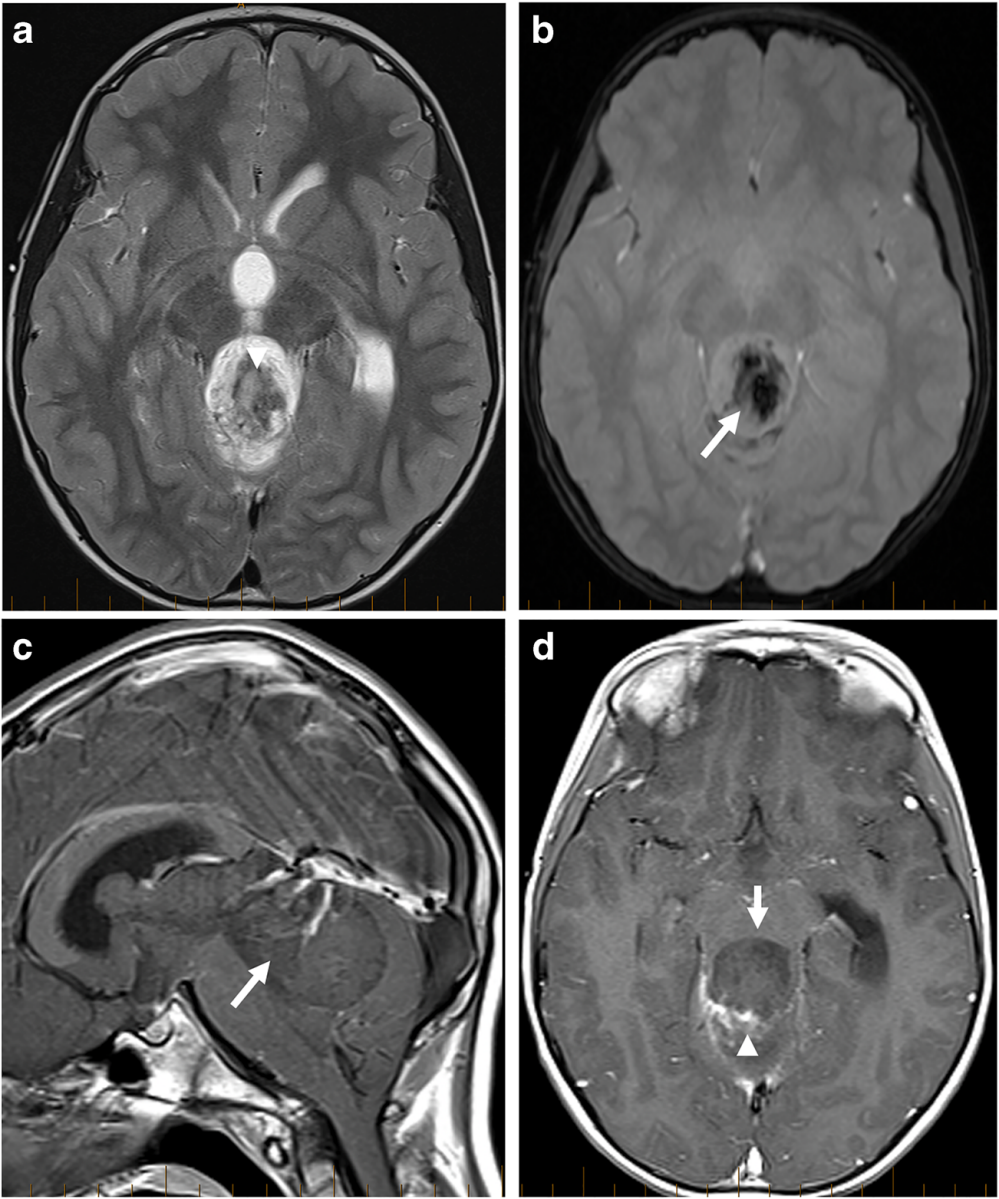
Typical imaging appearances of RGNT. The tumor involves the 4th ventricle and superior vermis and is heterogeneously hyperintense on T2-weighted sequence (**a**), mainly due to intratumoral hemorrhage that is better demonstrated on GRE images (arrow, **b**). Most of the tumor is nonenhancing (arrow, **c**, **d**), but areas of patchy enhancement (arrowhead, **d**) are seen on post-contrast T1-weighted sequences

Imaging: A classic RGNT is a heterogeneous-appearing mass in the fourth ventricle, typically having a cystic appearance, either solitary or multiple cysts with a solid component. The solid component of the tumor is isointense to hypointense in the T1-weighted sequence and hyperintense in the T2-weighted sequence and can demonstrate contrast enhancement [32, 65, 66]. Hemorrhage is common and may not be evident on CT scans [67] (Fig. 7). Calcification may also be present [67]. Satellite lesions have also been described [68]. As RGNTs arise closer to the aqueduct, they can present with obstructive hydrocephalus [69].

The solid component of the tumor does not demonstrate any diffusion restriction [65]. Low tumoral CBV has been reported on perfusion MRI [70]. An elevated choline peak with a mean choline-to-NAA ration of 1.86 has been reported on MRS [65].

#### PLGNTs arising from midline structures

As mentioned before, most midline PLGNTs are PAs, both in the posterior fossa and in the supratentorial compartment. Other tumors that are predominantly midline include pilomyxoid astrocytoma (PMA), SEGA, septal DNT, and RGNT. GGs and PDLGGs can arise from midline structures, predominantly in the basal ganglia and diencephalic regions [71]. Other histologic variants rarely arise from the midline structures [71], although midline structures can be secondarily involved in large hemispheric tumors. PLGNTs with FGFR1 alterations have a predilection to involve midline structures, particularly the diencephalon [5]. Also, (15:9) KIAA1549-BRAF fusion is more frequent in the midline PAs than in cerebellar PAs [16].

##### Tumors with the *KIAA1549–BRAF* fusion

**Pilocytic astrocytoma (PA)**

The A1549-BRAF fusion is the most common genetic alteration in midline PAs. Imaging appearances and histology of PAs arising from these sites are similar to those for cerebellar PAs. Only known site-specific genetic, imaging, and histomorphologic changes are enumerated here.

**Fig. 8:**
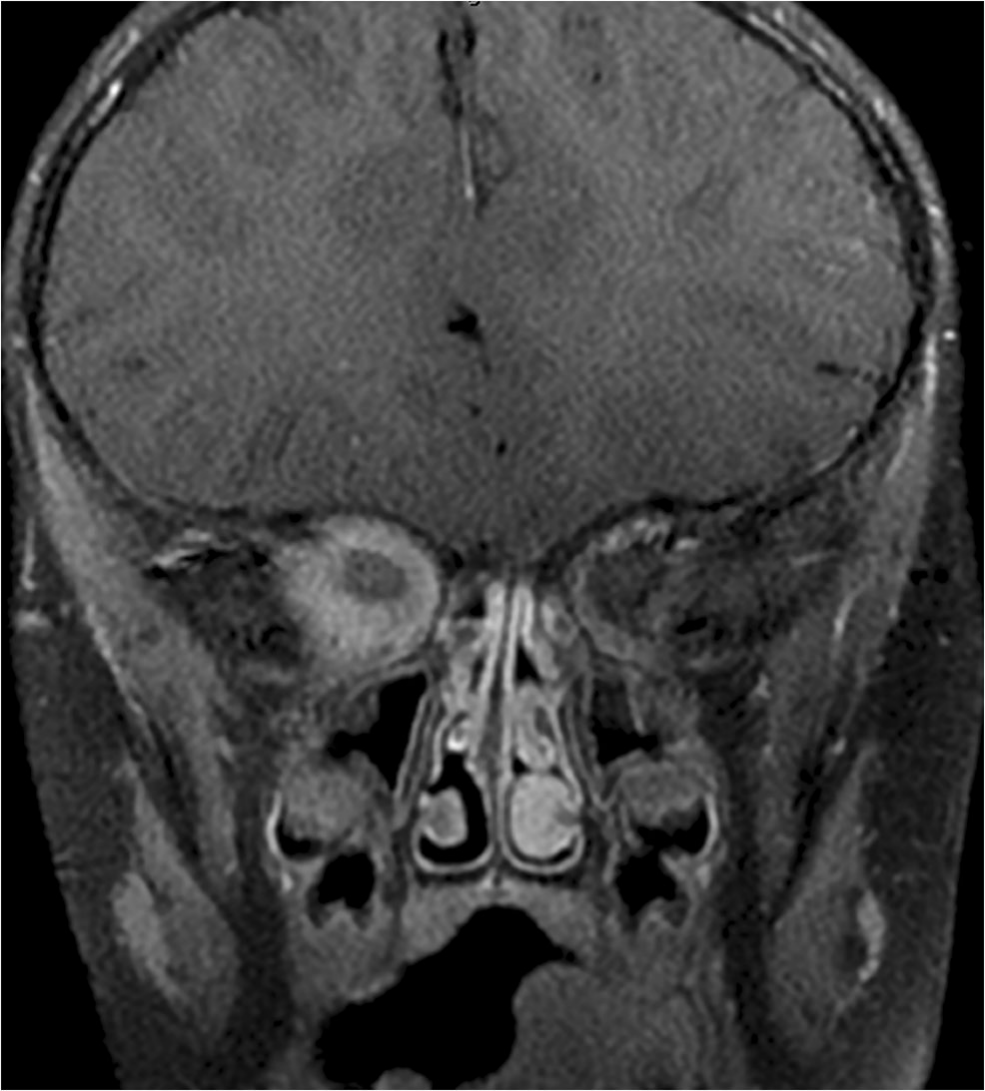
Optic nerve glioma mimicking optic nerve sheath meningioma. The postcontrast coronal T1-weighted image through the orbit shows the tumor around the central hypointense, minimally enlarged right optic nerve. There is no appreciable enhancement of the optic nerve proper. Minimal nerve enlargement may be the only difference between optic nerve glioma and optic nerve sheath meningioma

Optic nerves: PAs arising from the intraorbital optic nerves characteristically grow in the markedly expanded subarachnoid space between the optic nerve and the optic nerve sheath, with a well-delineated nerve structure in the middle of the tumor [13] (Fig. 8). This can sometimes be difficult to differentiate from optic nerve sheath meningioma.

**Fig. 9:**
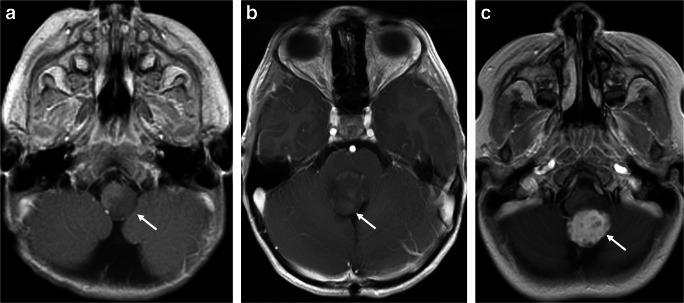
Enhancement patterns of dorsally exophytic brainstem glioma in three patients. **a** Axial post-contrast T1-weighted sequence through the medulla shows a nonenhancing (arrow) exophytic tumor arising from the left dorsal surface of the medulla in the first patient. **b** Axial post-contrast T1-weighted sequence through the pons in the second patient shows minimal patchy enhancement (arrow) of a dorsally exophytic tumor arising from the dorsal surface of the pons. **c** Axial post-contrast T1-weighted sequence through the medulla in the third patient shows an intensely enhancing (arrow) dorsally exophytic tumor arising from the dorsal surface of the medulla

Brainstem: Brainstem PAs generally originate from the dorsal surface and exhibit a dorsally exophytic growth pattern (Fig. 9) [72]. Radiologic appearances may be different from cerebellar PAs. Brainstem PAs often do, but occasionally may not, show any enhancement.

**Fig. 10:**
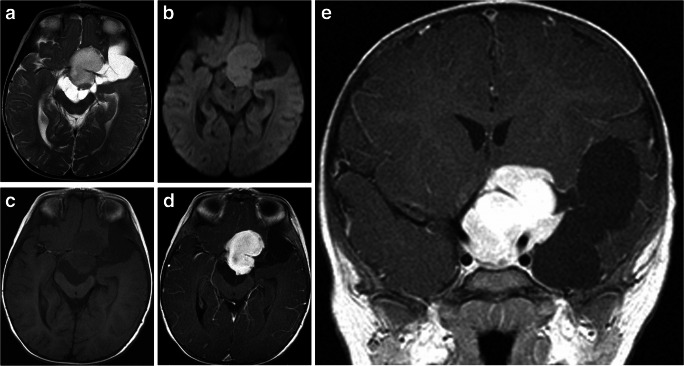
Pilomyxoid astrocytoma. **a** Axial T2-weighted sequence through the suprasellar region, demonstrating a large solid cystic tumor in the suprasellar region. The solid component is hyperintense compared to the gray matter and causes mass effects to the left frontal lobe. The amorphous cystic component conforms to the basilar cisterns, with splaying of the cerebral peduncles. **b** Axial diffusion image through the same level does not show diffusion restriction (ADC map not shown). **c** An axial pre-contrast T1-weighted sequence through the same level demonstrates that even the solid component of the tumor is hypointense compared to the adjacent gray matter. An axial post-contrast T1-weighted sequence through the same level (**d**) and the coronal T1-weighted sequence through the mid-sellar region (**e**) demonstrate intense enhancement of the solid component of the tumor. There is invasion of the left hypothalamus. The major arteries of the anterior circulation are surrounded by tumor on all sides. The cystic component extends into the left Sylvian fissure

Pilomyxoid astrocytoma (PMA), a subtype of PA, typically arises in young children (median age of presentation is 8 months) [73]. PMA almost always arises from the optic chiasm, hypothalamus, and adjacent brain tissues. PMA is characterized by a predominantly myxoid background and bipolar cells with angiocentric arrangement. The tumor is typically compact, with a non-infiltrative margin. Focal infiltration into the adjacent brain has been described. Vascular proliferation occurs in some cases, often as glomeruloid tufts. PMAs and PAs exist in a spectrum, and, over time, PMA can transition to PA in some cases [73]. The WHO has not assigned an official grade for this tumor because it is histologically benign but has higher rates of recurrence than does PA. The typical imaging appearance of a PMA is a well-circumscribed, T1-hypointense and T2-hyperintense mass centered in the hypothalamic/chiasmatic region, with a variable degree of contrast enhancement [74] (Fig. 10). Necrosis and invasion of adjacent brain areas are more common in PMAs than in PAs. Cyst and calcification are less common. In reported cases, PMAs demonstrate high choline and lipid/lactate peaks on MRS without any diffusion restriction [74].

##### Tumors with NF1 SNV

**Pilocytic astrocytoma (PA)**

CNS tumors develop in approximately 15–20% of children with NF1 SNVs; these tumors most commonly involve the optic pathway (in about 75–81% of cases) [8, 75]. These optic pathway tumors can arise anywhere along the optic pathway, from the intraorbital optic nerve to the optic radiations [75]. However, only ~50% of these tumors are symptomatic and ~33% of patients ultimately require therapeutic interventions [75]. Approximately 19–25% of PLGNTs in the NF1 setting arise outside the optic pathway: ~20% from the brain stem, ~5% from the cerebral hemispheres, ~5% from the cerebellum, and ~5% from the subcortical structures [8, 75].

Histology: In children, most tumors associated with NF1, whether arising from the optic pathway or the non-optic pathway, are low-grade gliomas, typically PAs, with low mitotic rates and proliferative indices [75]. The histology of these PAs is similar to that of PAs without NF1.

Imaging: As almost all PLGNTs associated with NF1 are of PA histology, imaging appearances of these tumors are similar to those of non-NF1 PAs, which are described above. Site-specific tumor characteristics are also similar. These tumors are typically indolent, with some completely regressing over time without any treatment [13]. Rarely, diffuse tumors can contiguously involve bilateral temporal lobes, basal ganglia, thalami, and varying degrees of cerebral hemispheres. These “deep extensive tumors” typically present at a young age (mean age, 3.9 years), have a worse prognosis, and need more aggressive treatment [76].

Frequently, other stigmata of NF1 occur alongside NF1-associated gliomas. Myelin vacuolization is one such NF1-associated CNS abnormality manifesting as ill-defined T2 hyperintensity in the deep brain nuclei and white matter of brainstem and cerebellum [77]. Myelin vacuolization is of particular importance, as it is often difficult to differentiate this feature from NF1-associated gliomas in imaging.

##### Tumors with PDGFRA mutations

**Septal dysembryoplastic neuroepithelial tumor (sDNT)**

In almost all cases, sDNT involves the septal nuclei/septum verum [78].

Histology: Typical sDNT tumor cells consist of oligodendroglia-like cells with perinuclear halo. The tumor cells frequently infiltrate the adjacent brain parenchyma that has mucinous matrix and microcysts. The glioneuronal element, the histological hallmark of cortical DNT, is characteristically absent in sDNT [78]. Mitotic figures are rare.

PDGRFA mutation is present in up to 78% of sDNTs. FGFR alteration is seen in only 11% of sDNTs, unlike in cortical DNT. In DNA methylation studies, sDNTs cluster differently from cortical DNTs and tumors with FGR1 alterations [78].

**Fig. 11:**
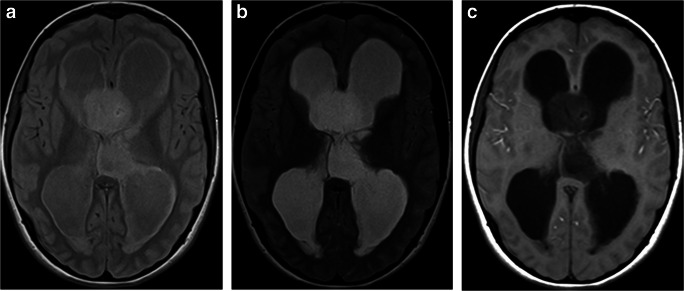
Septal dysembryoplastic neuroepithelial tumor. **a** An axial T2 FLAIR image through the level of the foramen of Monro demonstrates a minimally hyperintense mass causing obstructive hydrocephalus. **b** The mass is barely visible on the T2-weighted sequence because the signal intensity of the mass is similar to that of ventricular CSF. **c** The post-contrast T1-weighted sequence demonstrates no enhancement of the mass

Imaging: Similar to the cortical DNTs, the sDNTs have fluid signal (hypointense on T1-weighted sequence and hyperintense on T2-weighted sequence, with saturation of fluid signal on FLAIR sequence) on MRI images because of loose tumor matrix [78] (Fig. 11). Contrast enhancement is not a common imaging appearance and has been described in 15% of these tumors [78]. Typically, the tumor demonstrates elevated ADC value on diffusion imaging because of cystic matrix [78]. Calcification is typically absent, unlike in cortical DNT. Obstructive hydrocephalus is common because of the close vicinity of the tumor epicenter to the foramen of Monro [78].

##### Tumors with the TSC mutation

**Subependymal giant cell astrocytoma (SEGA)**

SEGA is found in patients with tuberous sclerosis, almost always around the foramen of Monroe.

Histology: SEGA is a well-circumscribed tumor composed of large plump astrocytes arranged in clusters, often with perivascular palisading. Tumor cells can exhibit a wide variety of appearances. The MIB1 proliferation index is usually low (<3%).

Tuberous sclerosis results from inactivating mutations of the TSC1 gene at chromosome 9q or the *TSC2* gene at 16p.

Imaging: The typical imaging appearance of SEGA includes a partially calcified, well-circumscribed mass in the subependymal region, most commonly around the foramen of Monro. On MRI, the tumor is heterogeneous on T1-weighted images and hyperintense on T2-weighted images, with a variable degree of contrast enhancement [79]. Prominent hypointensity may be seen in heavily calcified tumors. The presence of other CNS manifestations of tuberous sclerosis is critical to the radiological diagnosis of SEGA. Imaging is also critical to follow-up of SEGA.

##### Tumors with KRAS G12R SNV

**Tectal glioma (TG)**

Tectal glioma, by definition, arises from the tectal plate. Specific genetic and epigenetic features of TG suggest that this a distinct PLGNT phenotype.

Histology: Histologically, most TGs are similar to PAs. Few of them can have infiltrative margin similar to PDLGGs.

KRAS G12R is the dominant genetic abnormality in TGs with incidence rates as high as 82% [80]. Frequently, KRAS G12R SNV is concomitant with KIAA1549-BRAF fusion, BRAF pV600E SNV [80]. TGs form a distinct cluster on DNA methylation study. The difference in genetic alteration and methylation profile favors a distinct histogenesis of TGs and suggests this is a distinct entity [81].

Imaging: TGs may be relatively well-circumscribed but frequently extend beyond the tectal plate to the adjacent tegmentum and thalami [81]. Nodular enhancement can be present in 40% of cases [80]. A cystic component is rare. Because the tectal plate forms the roof of the aqueduct, the most constricted part of the ventricular system, obstructive hydrocephalus is very common (86%) [81].

#### PLGNTs arising from cerebral hemispheres

##### Tumors with the BRAF V600E SNV

**Ganglioglioma (GG)**

GGs typically arise from cortical/juxtacortical regions. GG is the most common tumor in the LEAT family [82]. It predominantly occurs in children and young adults and characteristically present with temporal lobe epilepsy.

Histology: GGs are well-differentiated, slow-growing tumors. The characteristic histology of GGs is a combination of dysplastic ganglion cells and neoplastic glial cells with marked heterogeneity. The GG spectrum can vary from a pure neuronal phenotype to a glial dominant phenotype. Usually, the neuronal component is dysplastic, and the glial component is proliferative. A fibrillary matrix is prominent. Myxoid degeneration or microcystic cavities may be present. Rarely, a dense capillary network can be seen within the solid component.

The most common genetic alteration in GGs is the BRAF V600E mutation, seen in up to 50% of cases. The KIAA1549-BRAF fusion occurs in up to 15% of cases.

**Fig. 12:**
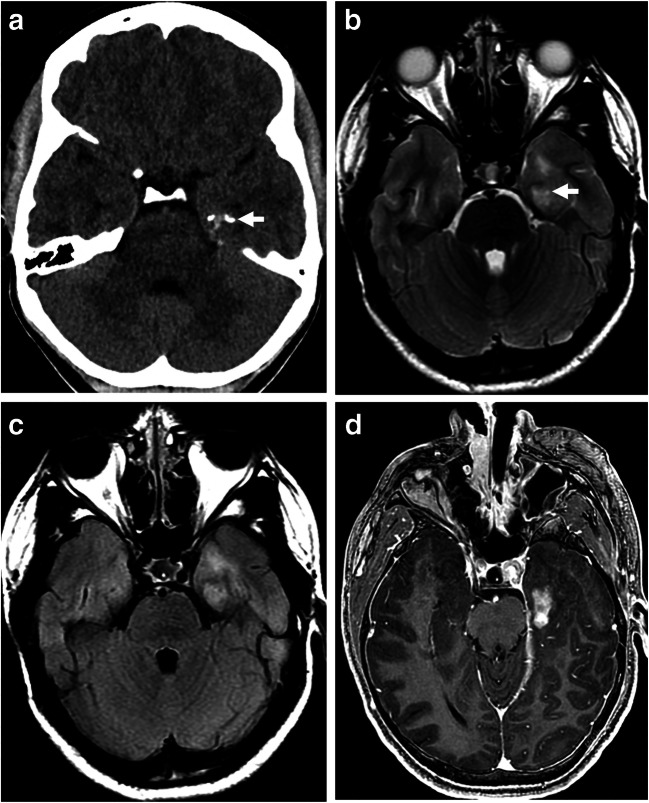
Ganglioglioma. **a** Axial non-contrast CT scan through the hippocampus, demonstrating a few punctate calcifications in the left hippocampus (arrow). No obvious tumor is identified. **b** An axial T2-weighted image through the same level, demonstrating ill-defined T2-hyperintense tumor (arrow) in the left hippocampus. The T2 abnormality extends to the left anterior temporal region. **c** An axial T2 FLAIR image through the same level better demonstrates the T2 abnormality in the left medial and anterior temporal lobe. Of note, there is no appreciable mass effect for the size of the tumor. **d** An axial postcontrast 3D T1-weighted image through the same level better shows heterogeneous enhancement of the hippocampal component of the tumor. The more anterior component does not show any abnormal enhancement

Imaging: Classic imaging findings include a cortical/juxtacortical mixed solid cystic or predominantly cystic tumor [83]. The temporal lobe is most commonly involved. The solid component demonstrates varying degrees of enhancement, from intense enhancement, to ring enhancement, to non-enhancement [83] (Fig. 12). Calcification can occur in up to 30% of tumors [83]. Because GG is a slow-growing tumor at the cortical or juxtacortical regions, scalloping of the adjacent inner cortex is a frequent finding, if involving the vertex [83]. Usually, little mass effect or edema is associated with this tumor. GGs with BRAF V600E mutation have lower ADC values than do GGs without the mutation [84]. However, GGs typically have higher ADC values than either low- or high-grade gliomas [85]. A high choline-to-NAA ration has been described on MRS [86]. Due to overlap of imaging appearances, differentiating cerebellar GGs from the cerebellar PAs can be challenging [87]. It has been reported that cerebellar GGs have lower relative ADCmin and higher relative CBV than PAs [88].

Pleiomorphic xanthoastrocytoma (PXA)

PXA is a superficial tumor that is almost exclusively limited to the supratentorial brain; cortical or juxtacortical regions of temporal lobes are the most commonly affected sites.

Histology: As the name suggests, these tumors demonstrate variable histological appearances. Spindle cells are intermingled with mononucleated or multinucleated giant astrocytes, with extreme variation in the size and staining of nuclei and frequent presence of intranuclear inclusion bodies. Additionally, there are large multinucleated cells with intracellular lipid droplets, which led to the designation “xanthoastrocytoma.” Despite a circumscribed appearance, most PXAs infiltrate the surrounding brain and extend into the perivascular Virchow–Robin spaces. The mitosis rate is typically <5%, and PXAs are classified as WHO grade II tumors. The proliferation index is variable in these tumor types and can be as high as 25% in WHO grade III anaplastic PXAs [89].

The BRAF V600E mutation occurs in up to 65% of all PXAs and up to 75% of WHO grade II PXAs [25, 90]. The BRAF V600E mutation in PXA, if present, is typically associated with younger age and worse prognosis [25, 90]. PXAs also frequently harbor a homozygous deletion of 9p21.3, which includes the tumor suppressor gene *CDKN2A*. Co-occurrence of BRAF V600E mutations with the CDKN2A deletion incurs poor prognosis because it increases the likelihood of dedifferentiation of PXAs to higher-grade gliomas, typically glioblastomas, as late as 10–20 years after the initial diagnosis [29].

**Fig. 13:**
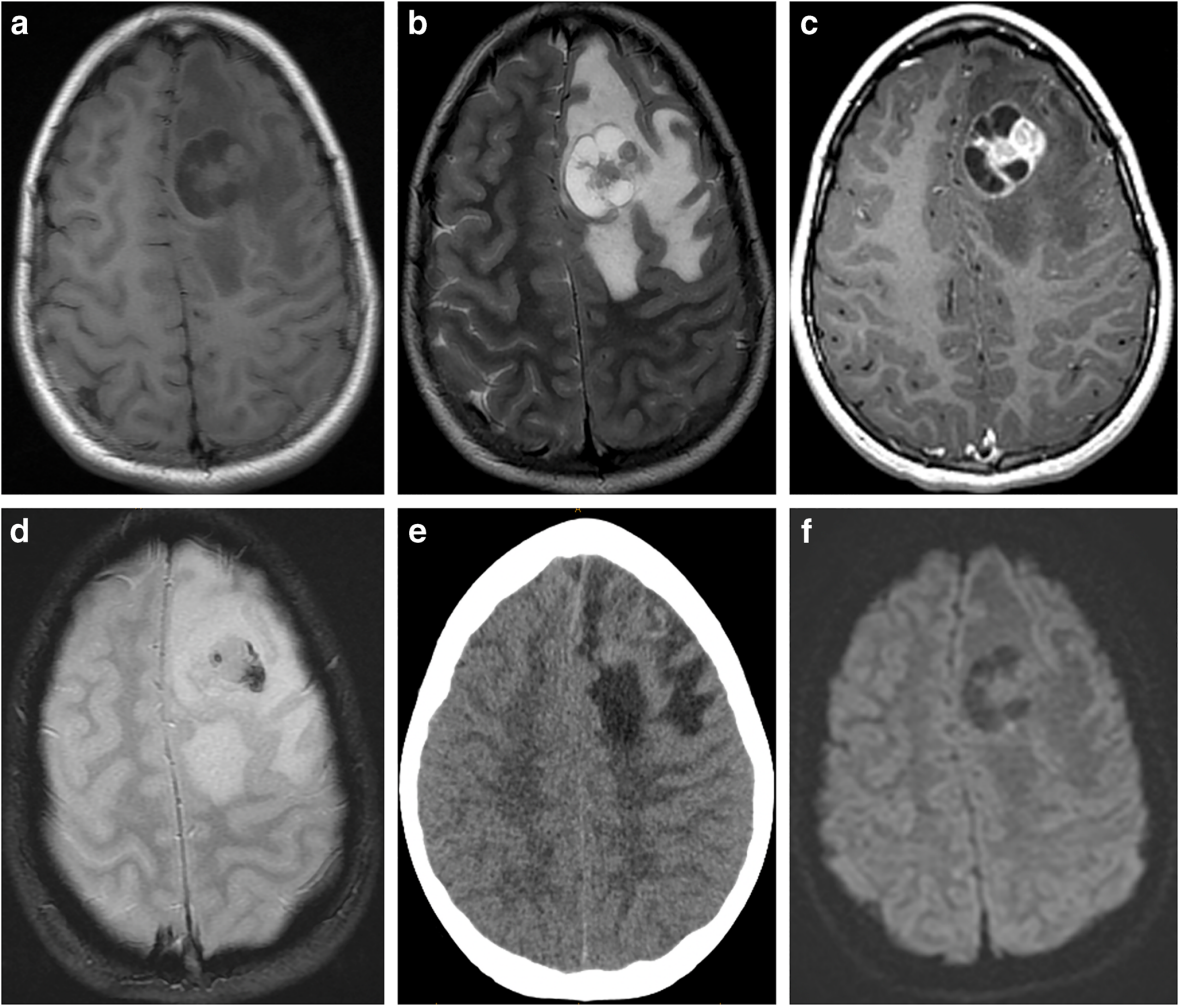
Pleiomorphic xanthoastrocytoma. **a** An axial non-contrast T1-weighted scan through the frontal lobes demonstrates a solid cystic tumor in the left frontal lobe. **b** Axial T2-weighted image through the same level better shows the solid cystic tumor. Extensive peritumoral T2 abnormality is also visible. **c** Axial postcontrast T1-weighted image through the same level better demonstrates enhancement of the solid component and the septae of the tumor. **d** Axial GRE image through the same level better demonstrates susceptibility artifact from intratumoral hemorrhage as there is no calcification within the tumor on CT (**e**). There is also no diffusion restriction on diffusion-weighted sequence (**f**)

Imaging: PXAs are superficial cystic tumors involving the supratentorial brain, occurring as either a cyst with a mural nodule or a tumor with multiple cysts, adjacent to the supratentorial leptomeninges. Leptomeningeal involvement can be seen in as many as two-thirds of the cases [91]. Calcification is rare. It is hypointense in the T1-weighted sequence and hyperintense in the T2-weighted sequence and shows intense contrast enhancement after contrast administration (Fig. 13). As this tumor is slow-growing, scalloping of the inner tables is seen. Significant perilesional edema is also a very frequent finding. Hemorrhage can also be present (Fig. 10). Diffusion MRI can show lower mean and minimum ADC values for the solid component than for GGs or PAs [92, 93].

Anaplastic PXAs demonstrate imaging appearance of more aggressive tumors compared to the PXA. Anaplastic PXAs are larger in size at presentation and have more obvious peritumoral edema, lower ADC value on DWI, and higher maximum relative CBV on MR perfusion imaging [94]. In adults, the enhancing areas of anaplastic PXAs demonstrate high choline-to-NAA ratio and lactate peaks MRS [95].

Desmoplastic infantile astrocytoma and desmoplastic infantile ganglioglioma (DIA and DIG)

DIA and DIG are cystic tumors of the superficial cerebral cortex with reactive changes in the adjacent leptomeninges (desmoplasia) in young infants (<2 years old); these changes usually arise from the frontal or parietal lobe [96].

Histology: Characteristic histologic findings include a desmoplastic leptomeningeal component and a poorly differentiated neuroepithelial component. Collagen deposition between cells is characteristic finding [97]. Astrocytic tumor cells are the sole tumor cell type in DIA. In DIG, neoplastic astrocytes are predominant along with the neuronal component, which is frequently dysplastic ganglion cells. In DIG, not in DIA, rests of primitive neuroectodermal ganglion cells, suggestive of anaplasia, are common [98]. Morphologically undifferentiated cell populations with small nuclei and scant cytoplasm with hypercellularity have also been described [99]. The proliferation index is variable but can be as high as 15% [100]. Some tumors can contain angiomatous vessels, but microvascular proliferation is rare.

Genetic architecture: BRAF SNV is very common in DIA and DIG. The BRAF V600E mutation is commonly seen in DIA, whereas a very rare form of BRAF SNV, BRAF pV600D mutation, is characteristically seen in DIG [98]. Regardless of the status of BRAF mutations, DNA methylation profiles indicate that DIA and DIG are different morphologic variants of single molecularly distinct entity [98].

**Fig. 14:**
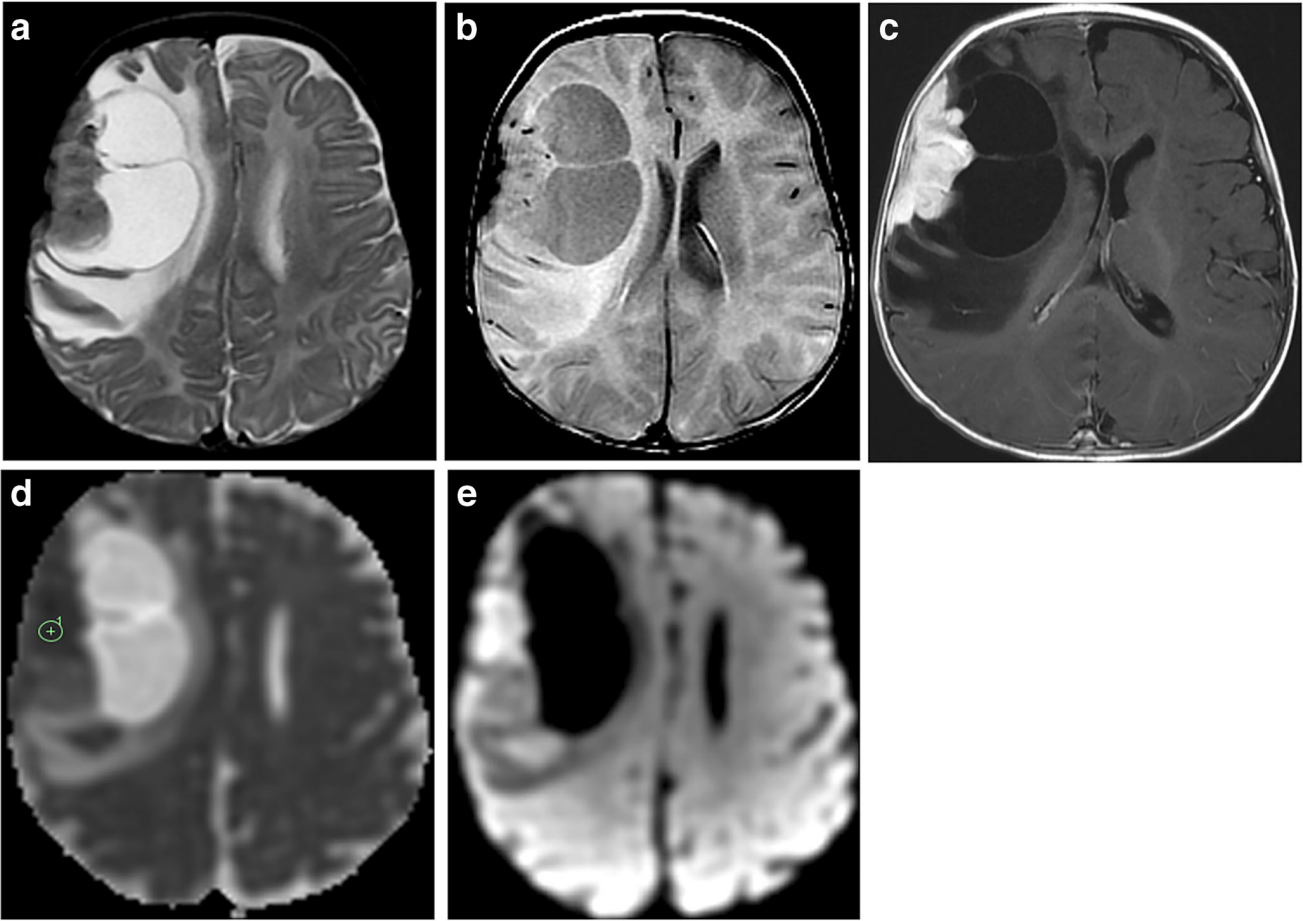
Desmoplastic infantile ganglioglioma. **a** Axial T2-weighted scan through the frontal lobes shows a large, peripheral, cystic tumor that has a large mural nodule at the leptomeningeal surface of the right frontal lobe. The nodule is hypointense compared to the adjacent gray matter. Moderate T2 abnormality extending well beyond the tumor margin that is also visible on axial T2 FLAIR image (**b**). **c** The post-contrast T1-weighted image demonstrates intense enhancement of the mural nodule that is attached to the leptomeningeal surface, a characteristic finding in this tumor. **d** The ADC map shows heterogeneous but low ADC values within the nodule (ADC_min_=497 × 10^−6^ mm^2^/s) and high value over the cystic component. **e** The DWI image demonstrates hyperintensity at the corresponding area of ADC_min_

Imaging: DIAs and DIGs are usually very large cystic tumors that can involve more than one lobe, most frequently the frontal and parietal lobes. Solid tumor without any cystic component has also been described [101]. The solid component is frequently located along the leptomeningeal surface. Although DIAs and DIGs are peripherally located tumors, due to their size, they are not limited to the cortical or juxtacortical region and frequently involve deep cerebral structures. The solid component is commonly hyperdense on CT images, hypointense on T2-weighted sequences, and demonstrates diffusion restriction, high perfusion, and intense contrast enhancement [91, 102] (Fig. 14). Absence of diffusion restriction on DWI has also been reported [103]. The ADC values of the DIAs and DIGs are lower than those of other low-grade gliomas because of restricted extracellular space secondary either to hypercellularity or to excessive collagen deposition [101]. The reported CBV within the solid component of the tumor is variable [104]. The enhancing area of the tumor has elevated choline, low NAA and low mI or lactate [105].

##### Tumors with FGFR1 rearrangement

**Dysembryoplastic neuroepithelial tumor (DNT; DNET is another commonly used acronym)**

DNT is another cortical/juxtacortical tumor in the LEAT family that typically involves the temporal lobe, with preferential involvement of the mesial temporal lobe, and typically presents with temporal lobe epilepsy [82].

Histology: The hallmark histologic finding in DNT is the multinodular growth pattern of glioneuronal elements, defined by columns of axon bundles lined by small oligodendrocyte-like cells perpendicular to the cortical surface. Between these columns, neurons with normal morphology are seen in a mucoid matrix.

The FGFR1 alterations are the genetic hallmark in DNTs, with FGFR1-TKDD fusion being the most common. FGFR1 SNV has also been described. Like other glioneuronal tumors, DNTs can harbor BRAF V600E mutations, usually in extra-temporal locations [39].

**Fig. 15:**
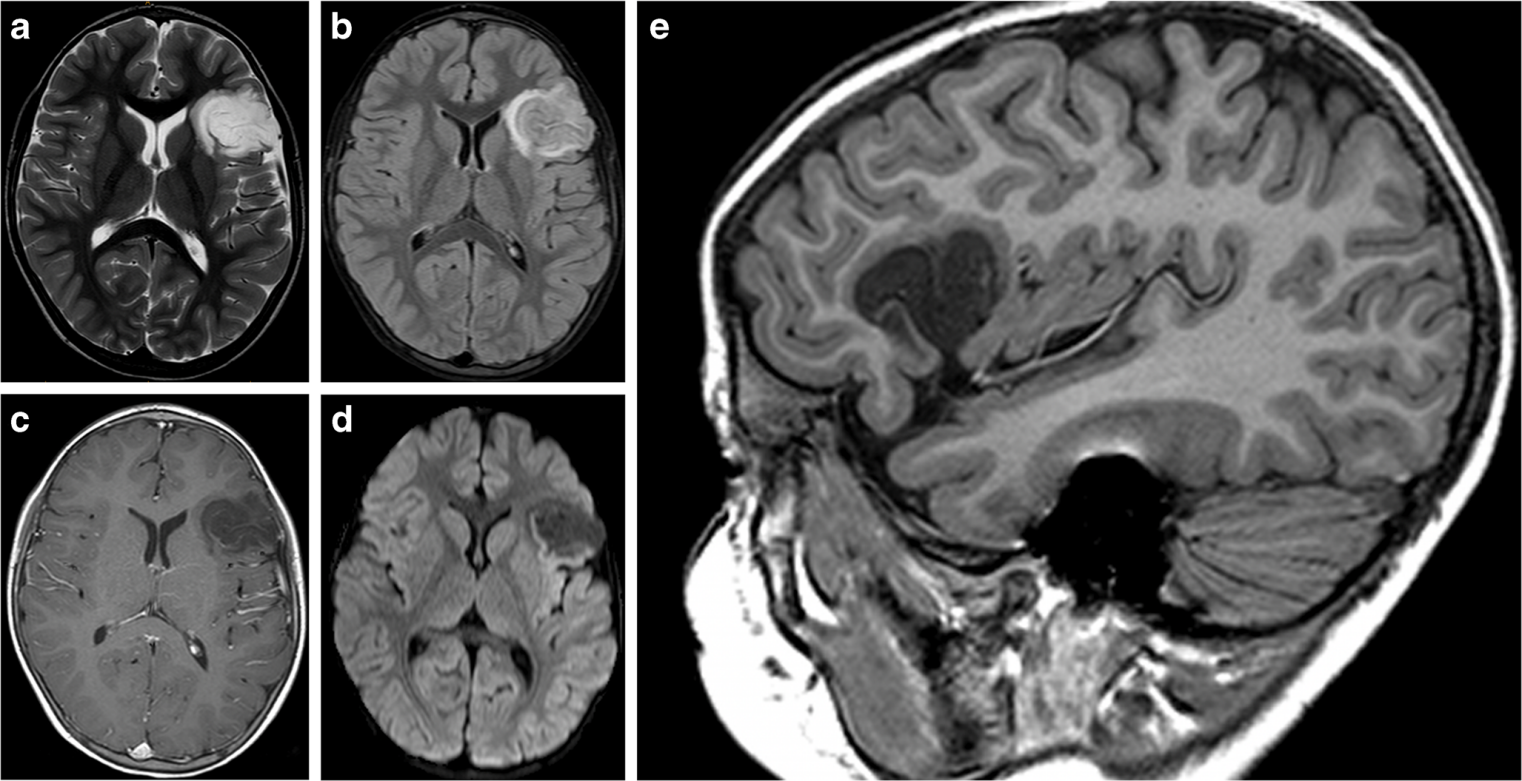
Dysembryoplastic neuroepithelial tumor. **a** An axial T2-weighted scan through the frontal lobes demonstrating a well-defined T2-hyperintense tumor in the left frontal lobe. **b** An axial T2 FLAIR image through the same level better demonstrates the internal architecture of the tumor; “mega-gyrus” appearance of the inferior frontal gyrus is better demonstrated. **c** Axial postcontrast T1-weighted image through the same level shows no enhancement. **d** Axial diffusion image through the same level shows no diffusion restriction. **e** Sagittal T1-weighted image better demonstrates the “mega-inferior frontal gyrus”

Imaging: DNT typically presents as an intracortical tumor without a significant mass effect [106]; smaller tumors can have a “mega-gyrus” appearance [91] (Fig. 15). It can have a multi-cystic appearance. In computed tomography images, the tumor manifests as a hypoattenuating mass that may occasionally have areas of calcification. DNT can have a broader area of involvement at the cortex, with a narrower apex pointing toward the ventricles, the so-called comet-tail appearance [107]. Remodeling of the adjacent inner table of the skull may also be seen, indicating the tumor’s indolent nature. The tumor is hypointense in the T1-weighted sequence and hyperintense in the T2-weighted sequence. Surrounding edema is characteristically absent. Contrast enhancement can be present in up to 33% of cases [91]. DNTs can be associated with distinct satellite lesions or a conglomerate of satellite lesions. This tumor typically demonstrates diffusion facilitation rather than restriction because of very loose matrix [102]. CBV of this tumor is lower than the adjacent normal cortex due to excessive water in the mucoid matrix [108]. On MRS, there is no significant differences in the choline-to-NAA, or choline-to-creatine peaks between the tumor and the normal brain [108]. Elevated mI peak has also been described on MRS [108].

##### Tumors with MYB/MYBL1 rearrangements

**Isomorphic diffuse glioma (IDG)**

IDGs arise from cortical or juxtacortical regions and are members of the LEAT family [82].

Histology: IDG typically has an isomorphic cellular growth pattern. The cellular density of this tumor type is typically low, with a loose matrix.

Half of the IDGs have copy number alterations of *MYBL1* or *MYB* genes [109]. Gene fusions of *MYBL1* or *MYB* with various gene partners are seen in the other half of IDGs [109].

**Fig. 16:**
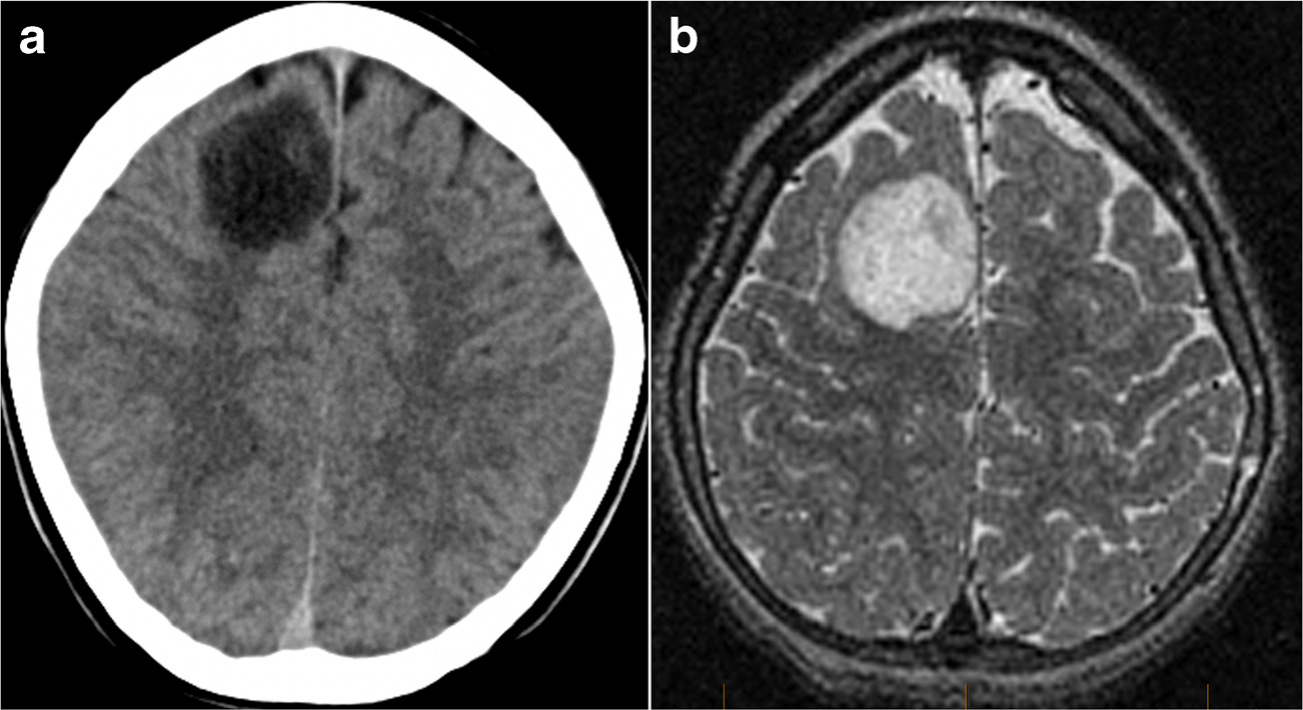
Isomorphic diffuse glioma. **a** Axial non-contrast CT scan through the frontal lobes shows a well-circumscribed hypodense tumor in the right frontal lobe. **b** Axial T2-weighted image through the same level demonstrates a well-circumscribed T2-hyperintense tumor

Imaging: IDG typically has a very well-circumscribed border and is more than 1 cm in diameter. Generally, this tumor is hyperintense in T2-weighted sequences and hypointense in T1-weighted sequences with no contrast enhancement [109] (Fig. 16). This tumor can be associated with cysts. Rarely, AG can arise from brain stem and may mimic diffuse infiltrating pontine glioma [110].

##### Tumors with *PRKCA* gene arrangement

**Papillary glioneuronal tumor (PGNT)**

PGNT is a rare glioneuronal tumor that occurs in the supratentorial compartment and is in the LEAT family [82]. Smaller PGNTs are primarily within the gray matter, but larger tumors extend into the white matter [111, 112].

Histology: PGNT is a dimorphic tumor having both neuronal and glial elements. The histologic hallmark of this tumor is the prominent pseudopapillary architecture of flattened or cuboidal glial cells with round nuclei and scanty cytoplasm, either in a single layer or pseudostratified, arranged around a hyalinized blood vessel intermingled with neurocytes and/or ganglion cells. The background can be fibrillary to microcystic. Hyalinized vascular structures can be prominent. This tumor does not exhibit microvascular proliferation.

The translocation of t (9;17) (q31;q24), resulting in the SLC44A1-PRKCA fusion oncogene, is the dominant genetic alteration, occurring in up to 90% of cases [5].

Imaging: PGNT is typically a hemispheric cystic tumor that has remarkably variable appearances and can mimic hemispheric PAs. PGNT can be purely cystic, a cyst with a mural nodule, a solid cystic tumor, or a completely solid tumor [113]. Intertumoral hemorrhage is common [114]. It is a well-circumscribed tumor and is typically devoid of any peritumoral edema, even when the tumor is very large. Leptomeningeal dissemination has not been reported. The solid component of the tumor typically demonstrates heterogeneous enhancement without any diffusion restriction or any hyperperfusion [114]. An elevated choline peak is the dominant finding on the MRS [114]. A lactate peak has also been described [114].

##### Tumors with MYB-QKI fusion

**Angiocentric glioma (AG)**

AG is another rare cortical/juxtacortical epilepsy-producing tumor that typically arises from the frontoparietal or temporal lobe. Similar to GGs, and DNTs, AG is a tumor of the LEAT family [82].

Histology: AG is usually a focal tumor that can have an infiltrative tumor margin. It is characteristically composed of monomorphous bipolar glial cells that are oriented around the cortical blood vessel, either along the length of the vessel or radially arranged around the vessel to form a pseudorosette-like ependymoma arranged along vascular structures. Some neurons may be entrapped in the infiltrative tumor component.

AG typically demonstrates MYB alterations with the MYB-QKI fusion being the genetic signature of this tumor [115].

**Fig. 17:**
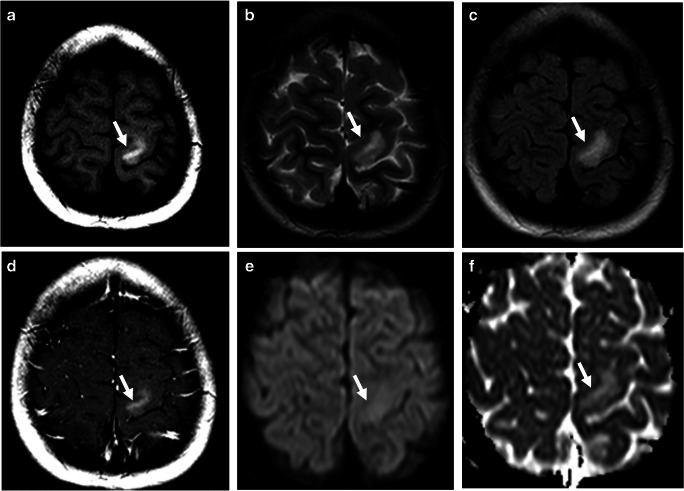
Angiocentric glioma. The tumor (arrows) is hyperintense on T1-weighted (**a**), T2-weighted (**b**), and T2 FLAIR (**c**) images, without appreciable enhancement on the post-contrast T1-weighted image (**d**). Also, there is no diffusion restriction as seen on the diffusion-weighted image (**e**) and ADC map (**f**)

Imaging: AG has a characteristic MRI appearance consisting of a ribbon-like cortical T1- and T2-hyperintense area with a handle-like extension toward the lateral ventricle (Fig. 17). AGs can also demonstrate a cystic component and can be difficult to differentiate from other superficial tumors [116]. The tumor does not enhance. Typically, this tumor does not have calcification, but calcification has been reported in rare cases [117]. Diffusion restriction is also atypical [118]. Although AG is primarily a focal cerebral hemispheric tumor, it can also arise from brainstem and may mimic diffuse infiltrating pontine glioma [110]. Markedly elevated mI and/or glycine, mildly elevated choline, and moderately decreased NAA have been described on single voxel MRS [119].

### Diffuse PLGNTs with no characteristic genetic alterations

#### PLGNTs arising from cortical/juxtacortical regions

##### Polymorphous low-grade neuroepithelial tumor of the young (PLNTY)

This tumor typically arises from the temporal lobe cortex or juxtacortical white matter of patients who have drug-resistant epilepsy, but it can also be found incidentally and may present with non-specific symptoms such as headache [120]. This tumor is not yet classified as LEAT [82].

Histology: This tumor shares many histologic features of oligodendroglioma and is *highly infiltrative* [5]. PLNTY is a polymorphous tumor with intertumoral and intratumoral heterogeneity. It can have a pseudo-rosette arrangement of cells [121]. Abundant calcification is the hallmark histologic feature of this tumor. This calcification can vary from discrete calcospherules to calcific masses with osseous metaplasia [121]. Myxoid microcysts and microvascular proliferation are notably absent [121] and MIB-1/Ki67 expression is negligible.

PLNTYs are associated with either BRAF-V600E SNV or FGFR alterations, in mutually exclusive pattern. BRAF-V600E SNV occurs in up to 40% of tumors; the FGFR alterations in the rest are either FGFR2 or FGFR3 fusions [122].

**Fig. 18:**
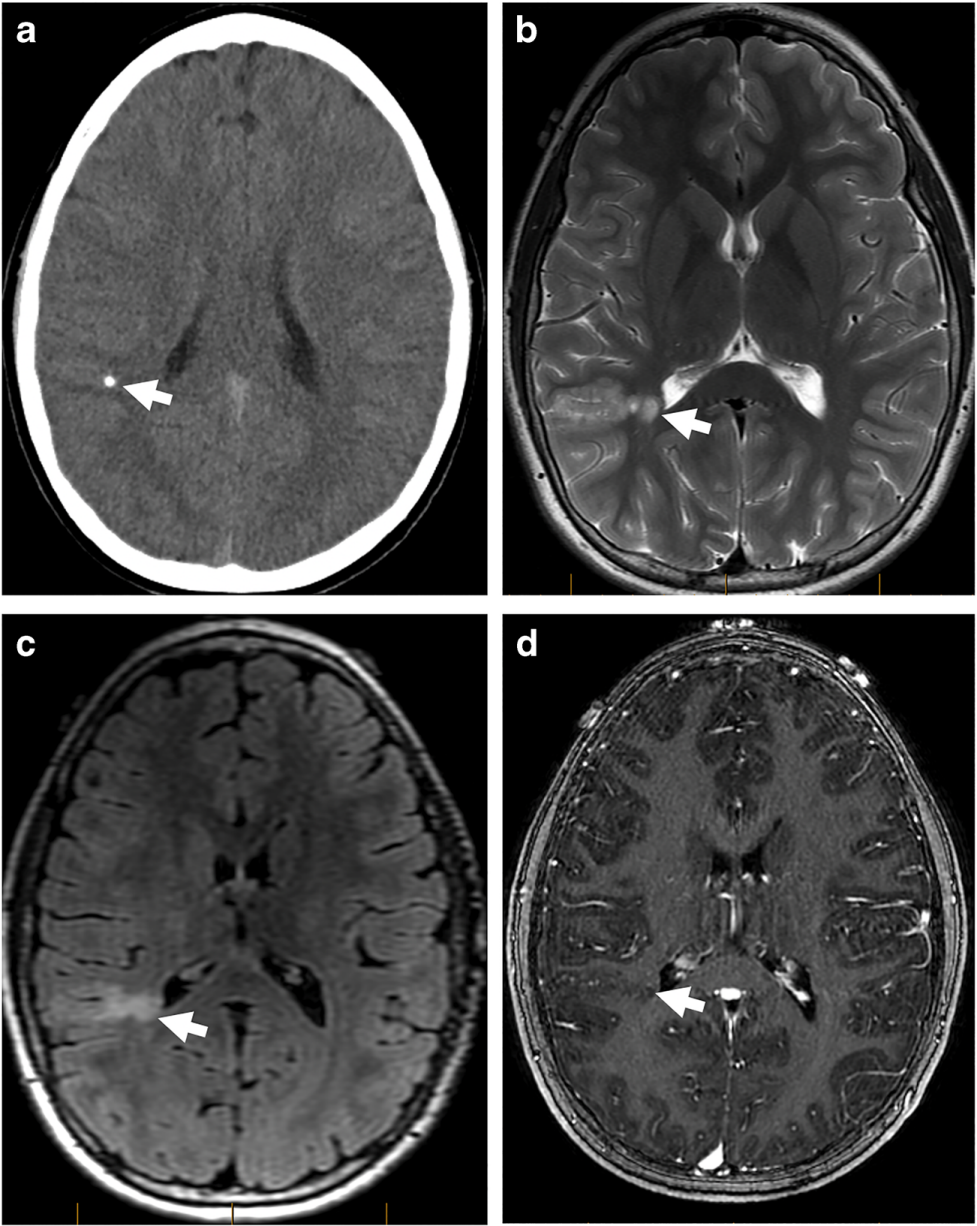
Polymorphous low-grade neuroepithelial tumor of the young. Macroscopic calcification (arrow) is characteristic on CT images (**a**). The tumor is hyperintense on T2 (**b**) and FLAIR (**c**), with no enhancement on the postcontrast T1-weighted image (**d**)

Imaging: This tumor is typically a round, well-circumscribed tumor showing heterogeneous tumor matrix on CT images and on different sequences on MRI. Most of the tumor has characteristic macroscopic calcification that is typically abundant at the center of the tumor [123] (Fig. 18). Peripheral cysts are very commonly found. PLNTYs can have calcifications and cysts. Restricted diffusion on DWI and high perfusion on perfusion imaging have been reported [123]. Imaging interpretation of diffusion and perfusion data should be done carefully, as abundant calcification might cause significant susceptibility effects on EPI-based diffusion and perfusion sequences.

#### PLGNTs arising from cerebral hemisphere

##### Pediatric-type diffuse low-grade gliomas (PDLGG)

PDLGGs constitute less than 10% of PLGNTs and predominantly involve the cerebral hemisphere white matter. Diencephalon and brainstem are less commonly involved areas [124].

Histology: Morphologically, PDLGGs have an astrocytic, oligodendroglial, or mixed oligo-astrocytic cellular lineage and may be difficult to distinguish from PAs, especially in small biopsies. Infiltration of the cerebral parenchyma is the defining histological character [7]. Mitotic activity is absent or very low; microvascular proliferation and necrosis are characteristically absent [7].

BRAF V600E SNV is the most common genetic alteration and can be present in up to 40% of PDLGGs. CDKN2A mutation can be concomitant with BRAF V600E SNV, and this combination is associated with poorer prognosis [124]. Two other common genetic alterations are MYB/MYBL1 structural alterations including amplification and FGFR1 alterations, either FGFR1 SNV or FGFR1-TKDD fusion. Gross total resection is not possible in many situations because of diffuse parenchymal involvement, so such tumors confer worse prognoses than do other PLGNT phenotypes. However, dedifferentiation to a higher-grade glioma is not a feature of PDLGG.

As mentioned in the histologic landscape section, PDLGGs are biologically different from adult diffuse low-grade gliomas, with a different disease course and prognosis even though histology and imaging of them are frequently similar.

Imaging: Although the biological difference between PDLGG and adult low-grade gliomas is known, literature on advanced imaging features of PDLGG is scarce. In our experience, imaging appearances of PDLGGs are similar to those of adult diffuse low-grade gliomas. PDLGGs are infiltrative tumors that predominantly involve cerebral hemispheres including deep cerebral structures. Typically, these tumors are ill-defined and hypointense on T1-weighted sequences and hyperintense on T2-weighted sequences. Diffusion restriction and contrast enchantment are rare. These tumors usually do not demonstrate any diffusion restriction or hyperperfusion. Elevated choline and low NAA are seen on MRS.

#### PLGNTs arising from leptomeninges

##### Diffuse leptomeningeal glioneuronal tumor (DLGNT)

DLGNT typically involves the leptomeninges of the spinal cord and posterior fossa. Leptomeningeal involvement of the supratentorial region can appear with disease progression.

Histology: On the basis of the methylation profile and histological and radiological appearances, DLGNTs can be classified into two methylation classes: MC1, MC2 [33]. DLGNT MC1 constitutes cells with round central nuclei with oligodendroglioma-line perinuclear halo in a desmoplastic stroma, whereas DLGNT MC2 is characterized by cells with round central nuclei without oligodendroglioma-like perinuclear halo.

Loss of chromosome 1p is the hallmark of DLGNTs and is present in all types of DLGNTs [33]. All DLGNT MC2 tumors also exhibit 1q gain. Co-deletion 1p/19q is frequent in DLGNTs, specifically in DLGNT MC1 tumors. Both subgroups can also harbor recurrent genetic alterations leading to MAPK pathway activation, with KIAA1549-BRAF fusion being the most frequent event [33].

**Fig. 19:**
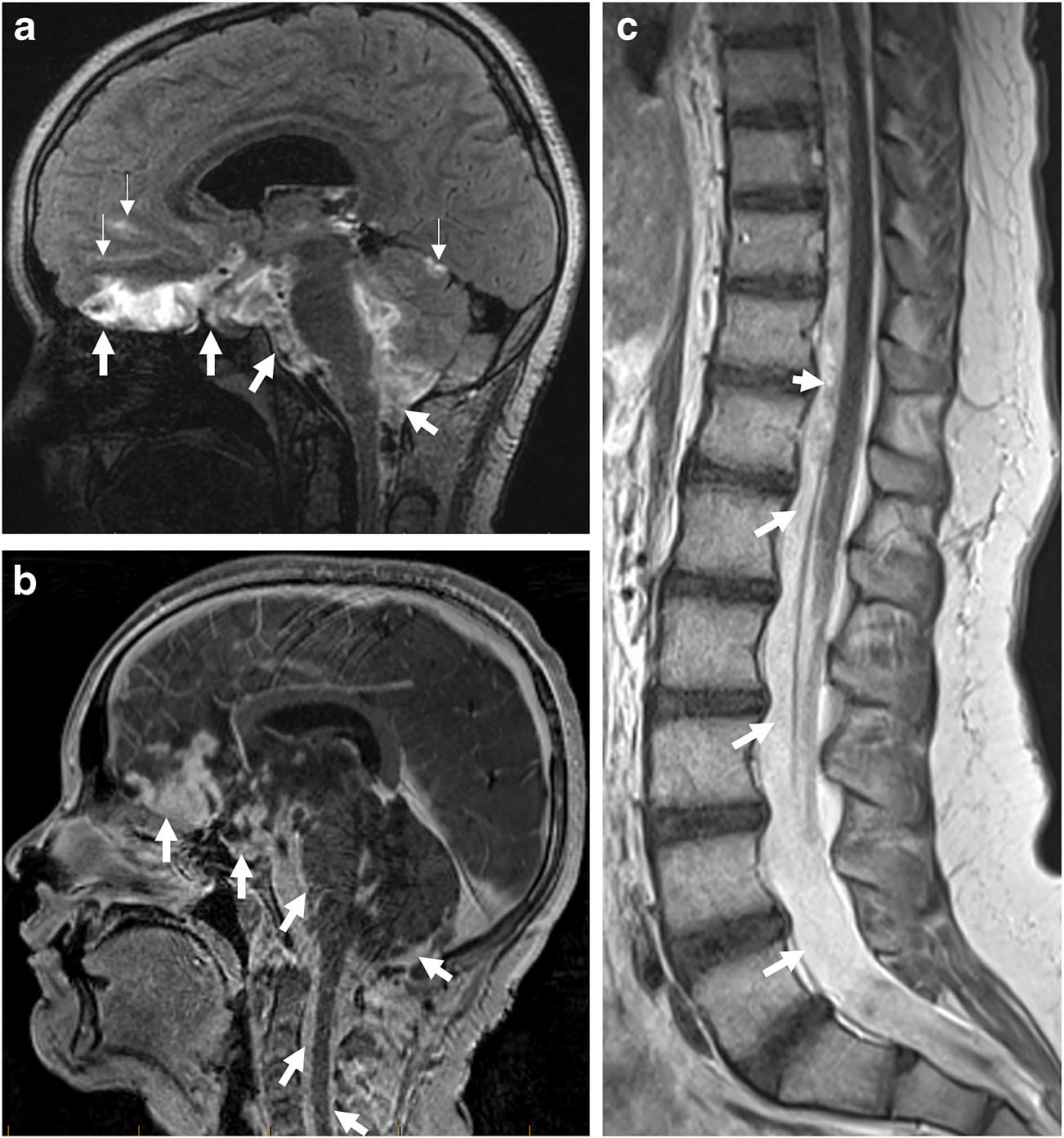
Diffuse leptomeningeal glioneuronal tumor*.* **a** Sagittal FLAIR image through the brain shows extensive FLAIR hyperintensity filling the floor of the anterior cranial fossa, basilar cistern, and the 4th ventricle (thick arrows). FLAIR hyperintensity is also present in the frontal lobe sulci and around the vermis (thin arrows). **b** Sagittal post-contrast T1-weighted image through the brain shows extensive amorphous enhancement of the leptomeningeal surface of the floor of the anterior cranial fossa, basilar cistern, and the 4th ventricle (thick arrows). **c** Sagittal post-contrast T1-weighted image through the lumbo-sacral regions shows extensive enhancement of the CSF space surrounding the cord and the cauda equina nerve roots (arrows)

Imaging: The typical imaging appearance of DLGNT is an intramedullary enhancement of the spinal cord and enhancement of the leptomeningeal surface of the cord, with occasional enhancement of the posterior fossa leptomeninges [33]. As the disease progresses, leptomeningeal enhancement around the cerebral hemispheres ensues [33] (Fig. 19). Cystic changes within parenchyma adjacent to the abnormal leptomeninges are also common. Spinal leptomeningeal enhancement is more common in MC1 (93%) than in MC2 (58%). Leptomeningeal enhancement at presentation or evidence of radiologic dissemination can be absent, and leptomeningeal enhancement on MRI is not an essential criterion for diagnosing DLGNT [33].

## Conclusion

Activation of the MAPK signaling pathway is a singular event that drives the development of PLGNTs, especially those due to alterations in the *BRAF* gene. The KIAA1549BRAF fusion and BRAF pV600E SNV are the most common BRAF alterations in PLGNTs. *NF1* is the second most commonly affected gene in the MAPK pathway followed by FGFR1. PLGNTs not associated with MAPK pathway activation are rare. Specific genetic alterations influence not only tumor histologic phenotype but also tumor location and imaging appearance (e.g., cyst formation, enhancement, hyperperfusion), features which are extremely important for a neuroradiologist’s proper imaging assessment of the individual PLGNT phenotype. Genetic alterations also influence the prognosis of a specific PLGNT phenotype. The interaction of genetics and histology on imaging and on prognosis is reviewed in detail. A radiohistogenome-based stratification scheme of this extremely heterogeneous group of tumors is described here, which is in alignment with the current stratification scheme used for management.

## Data Availability

Not applicable
